# Supplementary material for: Fundamental equations linking methylation dynamics to maximum lifespan in mammals
Source: Nat Commun. 2024 Sep 16;15:8093. doi: 10.1038/s41467-024-51855-z (PMC11405513; doi:10.1038/s41467-024-51855-z)
Supplement: Supplementary file 1 — Supplementary Information [file 41467_2024_51855_MOESM1_ESM.pdf]

Supplementary Information

For the article (Horvath et al. (2024) Fundamental Equations Linking Methylation Dynamics to Maximum Lifespan in Mammals.), the file contains Supplementary Figures 1 - 19, Supplementary Notes 1 - 3, and the legends for Supplementary Data/Tables.

Supplementary Figures

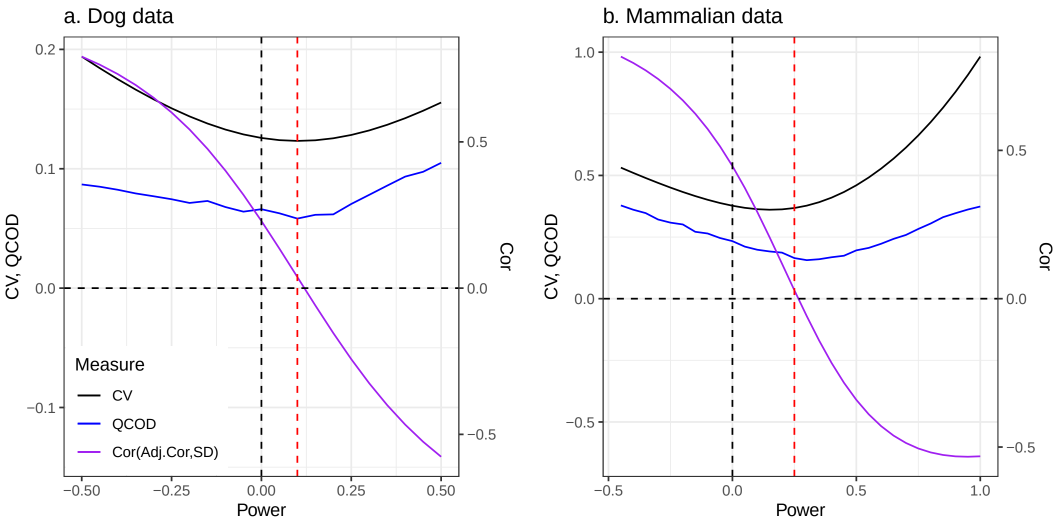

Supplementary Fig. 1: **Optimal power for Adj.ROC in (a) Dog data and (b) Mammalian data.** The x-axis reports the adjustment power p used in our definitions of adjusted Pearson correlation and adjusted AROCM. The CV (Coefficient of Variation equation 10), Quartile Coefficient of Dispersion QCOD (equation 26), and $\mathrm{Cor}(Adj.\mathrm{Cor}, \mathrm{SD}\,(R))$ are plotted versus different values of the power p. The optimal power (dashed red vertical line) is chosen to be 0.1 for the dog data and 0.25 for the mammalian data.

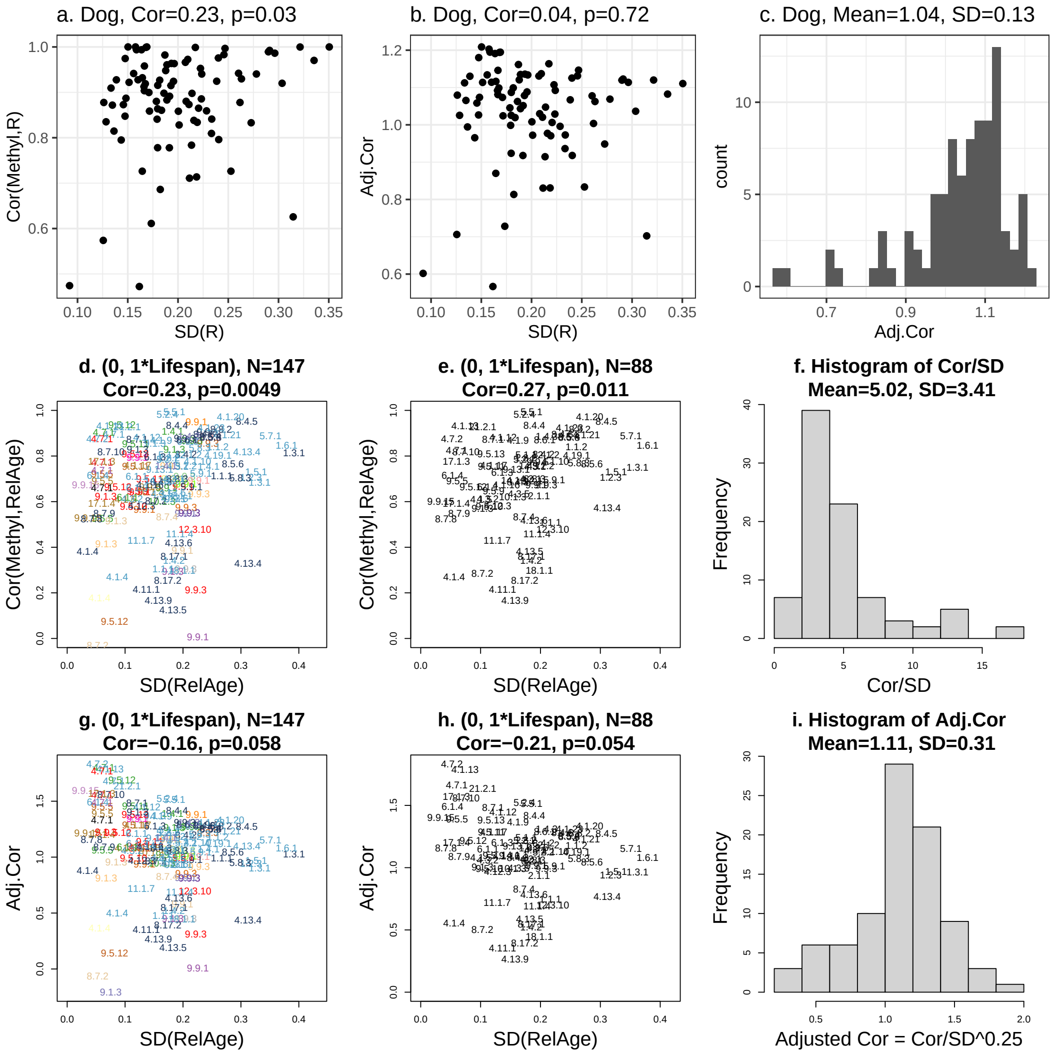

Supplementary Fig. 2: **Age Correlation versus Standard Deviation of Relative Age. (a-c)** dog data, $N = 90$ breeds; **(d-i)** mammalian data. **(a,d,e)** age correlation $\text{Cor}(M, A)$ and **(b,g,h)** the adjusted age correlation *Adj.*Cor against the standard deviation of relative age SD $(\mathbf{R})$. Histograms of **(c)** Adj.Cor in dogs, **(f)** Cor/SD in mammals, and **(i)** Adj.Cor in mammals are also provided. Titles of scatter plots report Pearson correlation coefficients and associated nominal (uncorrected) two sided Student T test p-values. N denotes the sample size. The fact that the adjusted age correlations result in insignificant Pearson correlation test p-values with SD (R) suggests that these adjusted correlations are less susceptible to biases in sample ascertainment.

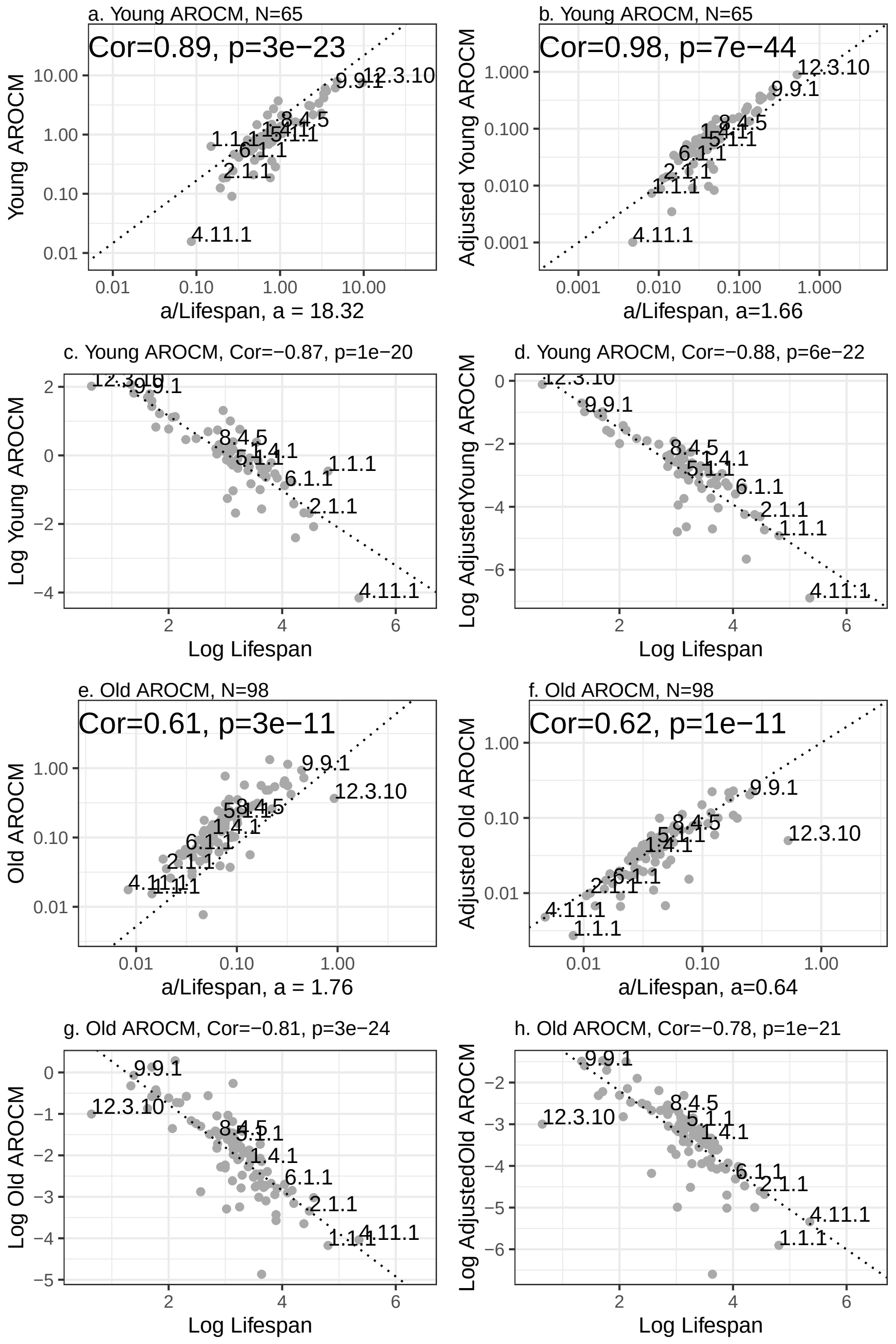

Supplementary Fig. 3: **Young and Old AROCM versus max lifespan.** This figure is analogous to Figure 5 but with Young and Old AROCMs. **a-d.** the sample size for Young AROCMs is $N = 65$; **e-h.** the sample size for Old AROCMs is $N = 98$. Titles of the scatter plots report Pearson correlation coefficients and associated nominal (uncorrected) two sided Student T test p-values.

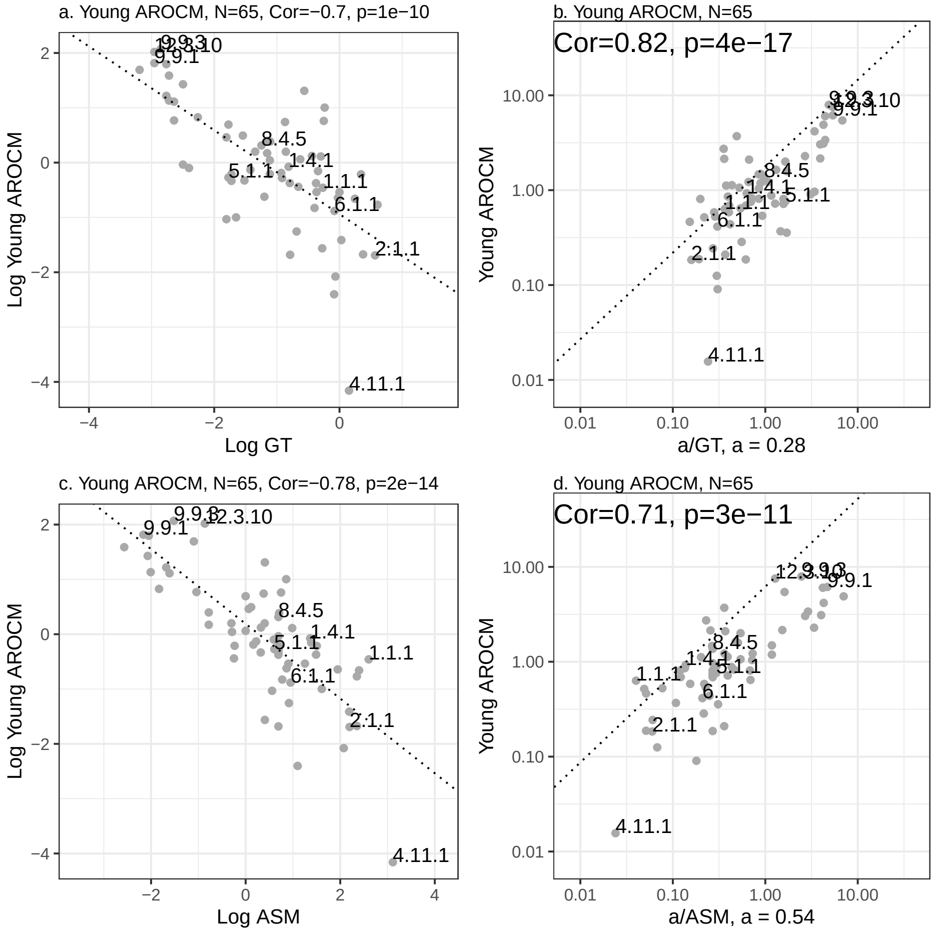

Supplementary Fig. 4: **AROCM in young animals versus gestation time**. Young AROCM$[0, 0.1L]$ vs. Gestational Time (GT, **panels a,b**) and Age at Sexual Maturity (ASM, **panels c,d**). Titles of the scatter plots report Pearson correlation coefficients and associated nominal (uncorrected) two sided Student T test p-values. N denotes the sample size.

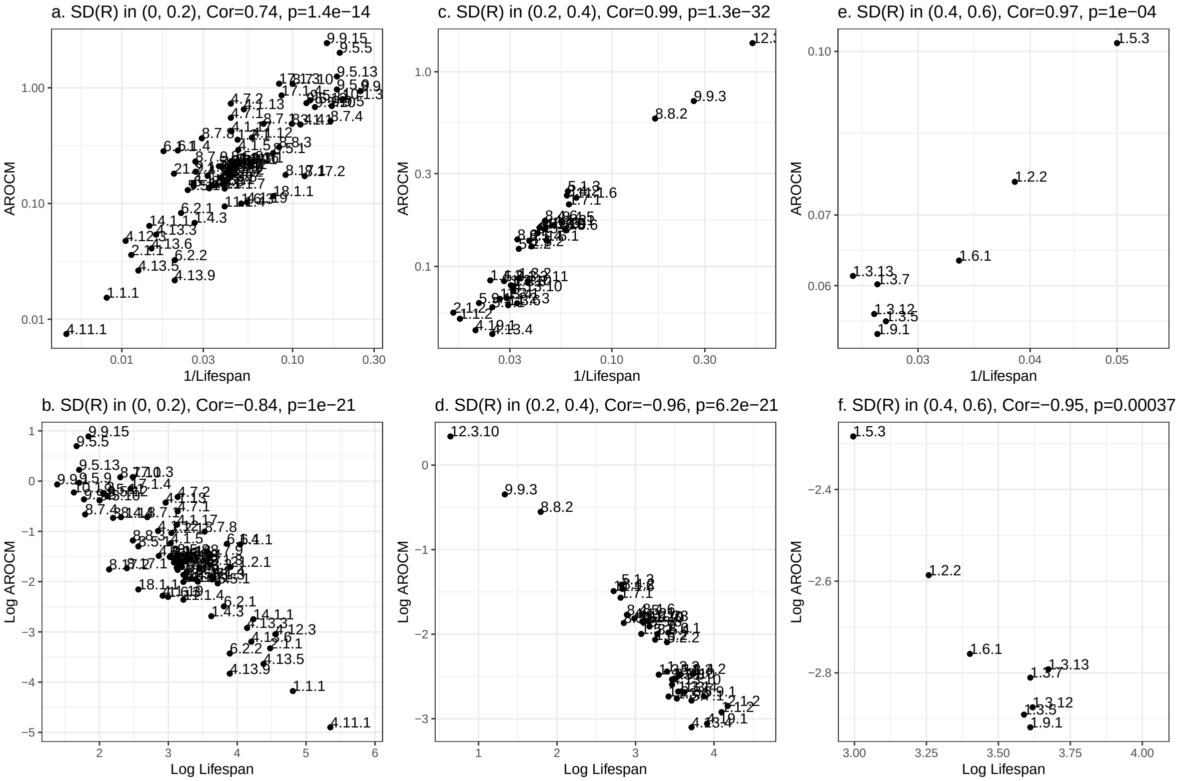

Supplementary Fig. 5: **AROCM versus max lifespan by similar** $\mathrm{SD}\,(R)$ **values in mammalian data.** This figure is analogous to Figure 5 but groups the strata by their empirical standard deviation of relative ages. **Panels a,c,e** AROCM vs. 1/Lifespan; **b,d,f** Log(AROCM) vs. Log(Lifespan). **a,b** SD(R) in (0,0.2); **c,d** SD(R) in (0.2,0.4); **e,f** SD(R) in (0.4,0.6). Titles of the scatter plots report Pearson correlation coefficients and associated nominal (uncorrected) two sided Student T test p-values. N denotes the sample size.

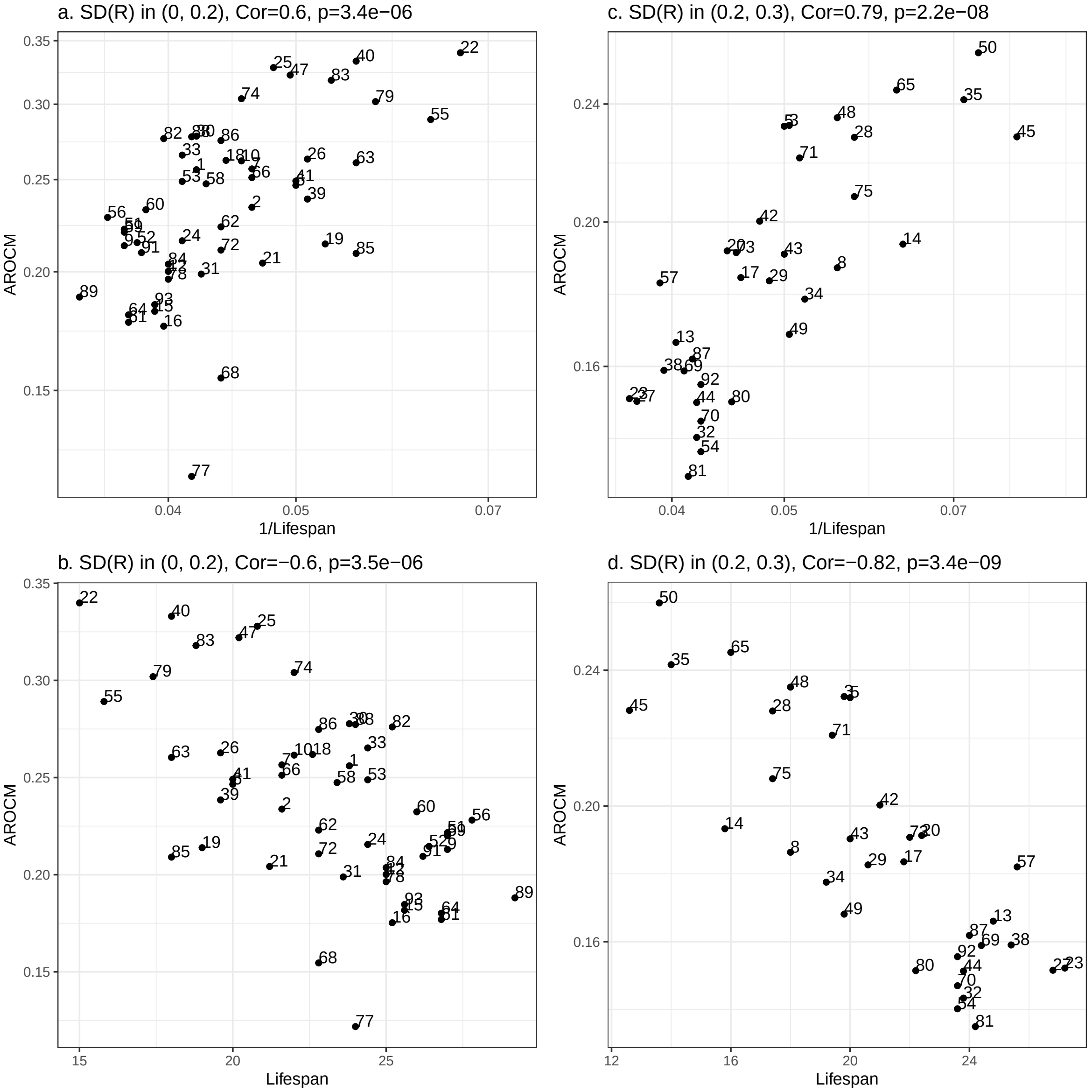

Supplementary Fig. 6: **AROCM versus max lifespan by similar** $\mathrm{SD}\,(R)$ **values in dog data.** This figure is analogous to Figure 1a,b,e,f, but groups the strata by their empirical standard deviation of relative ages. **Panel a.** AROCM vs. 1/Lifespan in dog breeds with SD(R) in (0,0.2); **b.** AROCM vs. Lifespan in dog breeds with SD(R) in (0,0.2); **c.** AROCM vs. 1/Lifespan in dog breeds with SD(R) in (0.2,0.3); **d.** AROCM vs. Lifespan in dog breeds with SD(R) in (0.2,0.3). Titles of the scatter plots report Pearson correlation coefficients and associated nominal (uncorrected) two sided Student T test p-values. N denotes the sample size.

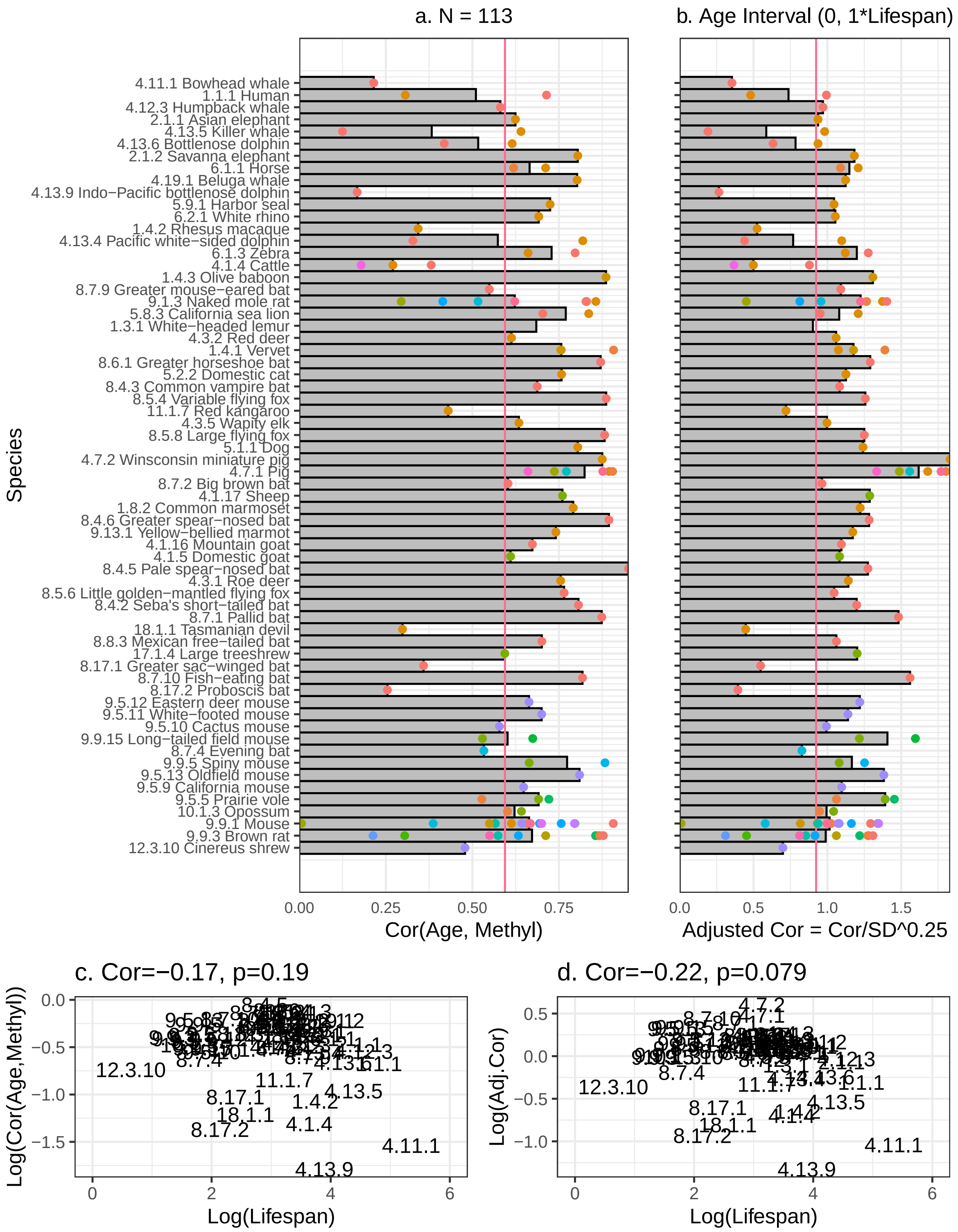

Supplementary Fig. 7: **Species level analysis shows that age correlations do not relate to maximum lifespan. Panels a, b:** Barplots for Cor$(Age, Methyl)$ and $Adj.$Cor $= \frac{Cor(Age,Methyl)}{\text{SD}\,(\mathbf{R})^{0.25}}$ in different species (113 species tissue strata with at least 15 samples). Each horizontal bar (y-axis) corresponds to a different species. Species are sorted by maximum lifespan: from long lived (top) to short lived (bottom). Each bar length reports correlation value for each species across all tissue types, while the dots report the values for different tissue types. The red vertical bars show the mean values of correlations in the respective panels. The QCOD for Cor$(Age, Methyl)$ is 0.189 and that for $Adj.$Cor is 0.137. Mean and Median Cor$(Age, Methyl)$ across species are 0.66 and 0.69 respectively, and those for $Adj.$Cor are 1.04 and 1.12. Following equation (1), Methyl was defined as mean methylation value across all CpGs located in bivalent promoter state 2 (BivProm2+). The Pearson correlation is -0.166 (nominal two sided Student T test p=0.19) for Cor$(Age, Methyl)$ in **panel c** and -0.22 (two sided p = 0.08) for $Adj.$Cor in **panel d**.

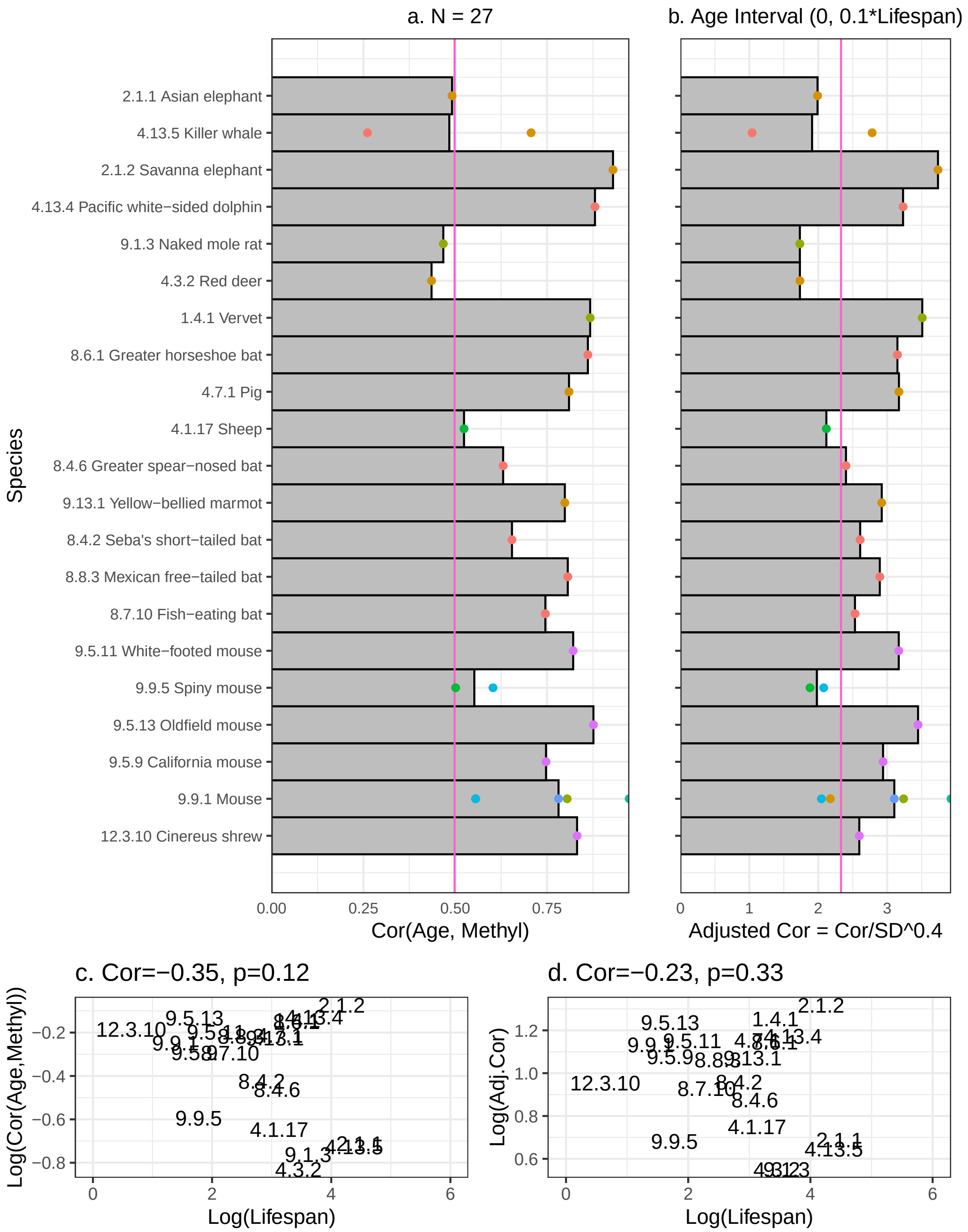

Supplementary Fig. 8: **Species level age correlations in young animals**. This analysis, akin to Supplementary Fig. 7, focuses on younger animals with relative age $R < 0.1$, and species are arranged by lifespan. Each dot represents a different tissue. **Panel a** displays Cor(Age, Mean Methylation in BivProm2+) for strata with sample size >= 15; **panel b** shows Adj.Cor for the same strata. The Mean, Median, and QCOD for Cor(Age,Methyl) are 0.5, 0.6, and 0.137 respectively; for Adj.Cor, they are 2.3, 2.6, and 0.115. The Pearson correlation is −0.35 (p=0.12) for Cor($Age, Methyl$) in **panel c** and −0.23 (p = 0.33) for $Adj.$Cor in **panel d**. Titles of the scatter plots report Pearson correlation coefficients and associated nominal (uncorrected) two sided Student T test p-values. N denotes the sample size.

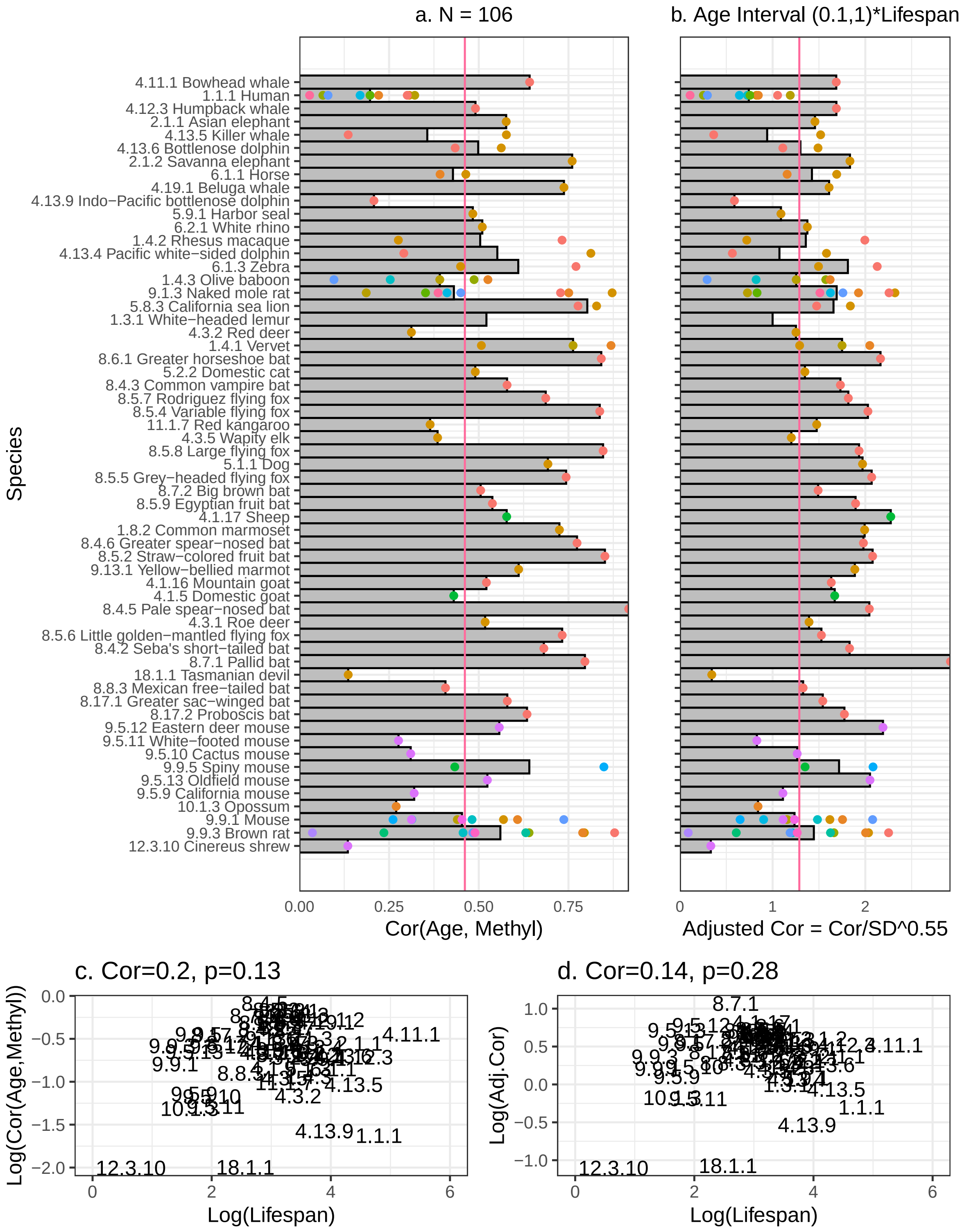

Supplementary Fig. 9: **Species level age correlations in old animals**. This analysis mirrors Supplementary Fig. 7, focusing on older animals with relative age $R \geq 0.1$, and species are sorted by lifespan. Each dot signifies a different tissue. **Panels a,c** show Cor(Age, Mean Methylation) in BivProm2+ for strata with sample size >= 15; **Panels b,d** show Adj.Cor for the same strata. The Mean, Median, and QCOD for Cor(Age,Methyl) are 0.46, 0.51, and 0.389 respectively, and for Adj.Cor, they are 1.17, 1.39, and 0.253. Titles of the scatter plots report Pearson correlation coefficients and associated nominal (uncorrected) two sided Student T test p-values. N denotes the sample size.

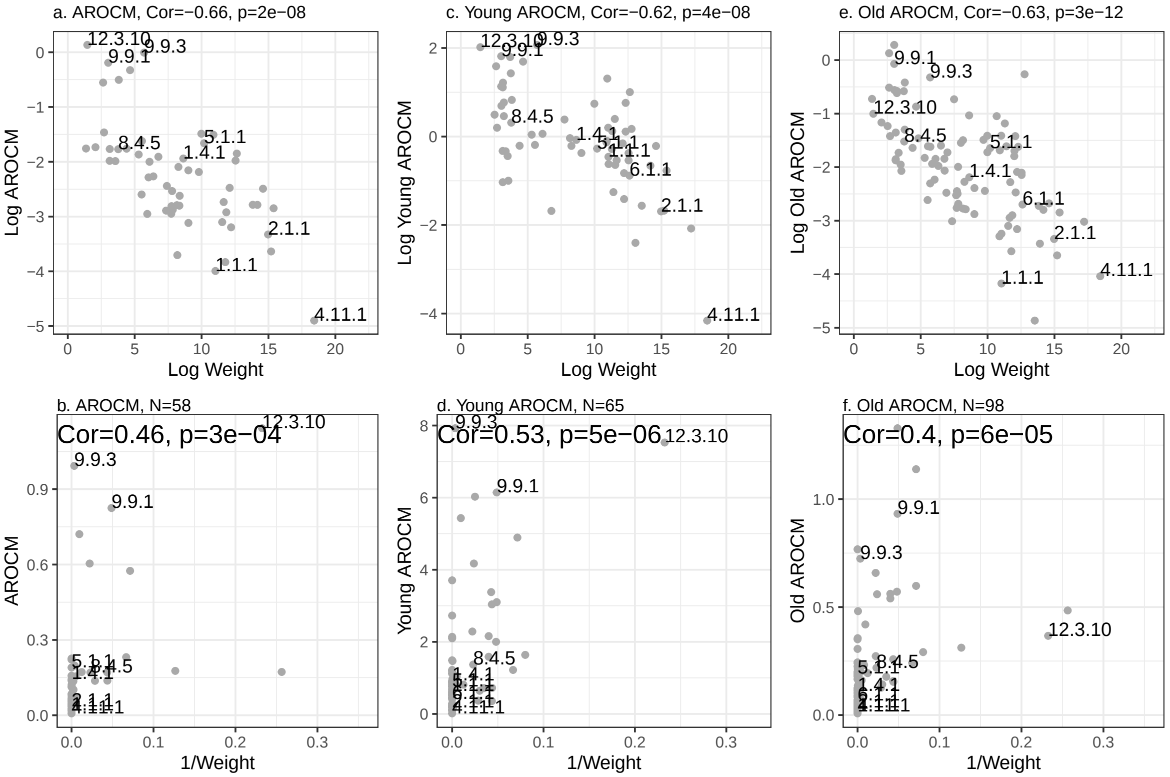

Supplementary Fig. 10: **AROCM versus average adult weight in different age groups**. Columns correspond to different age ranges: **(a,b)** all ages AROCM, **(c,d)** Young AROCM$[0, 0.1L]$, and **(e,f)** old AROCM. The first row panels **(b,c,e)** present log transformed AROCM (y-axis) against average adult weight (x-axis). The second row panels **(b,d,f)** contrast untransformed AROCM values (y-axis) with $1/Weight$ (x-axis) Titles of the scatter plots report Pearson correlation coefficients and associated nominal (uncorrected) two sided Student T test p-values. N denotes the sample size.

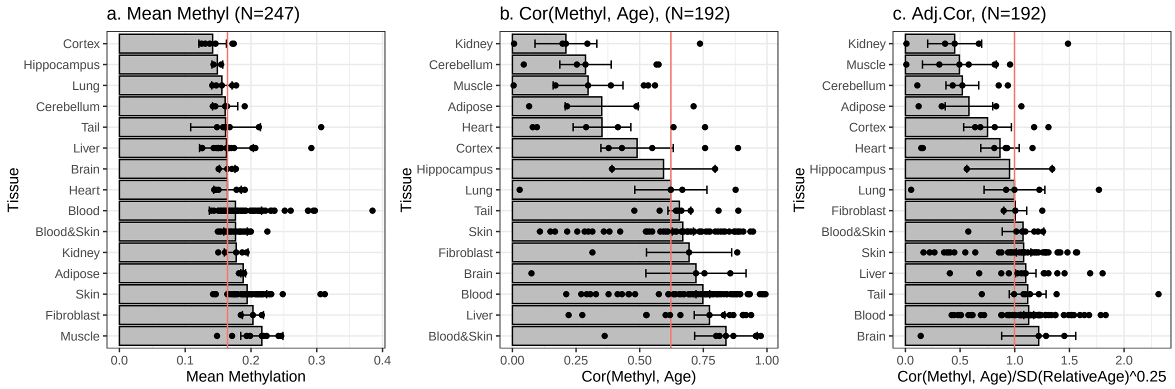

Supplementary Fig. 11: **Age correlations in different mammalian tissue types.** This figure depicts Mean Methylation (**Panel a**), Cor(Methyl, Age) (**Panel b**), and Adjusted Cor (**Panel c**) in BivProm2+ across tissue types. Error bars signify 1 standard error from the median per tissue type, with the red vertical line indicating the overall median. Results, however, are confounded by species, prompting the use of multivariate regression models to explore tissue type effects (Table 1). The number of species within each tissue can be found in Supplementary Data 3.

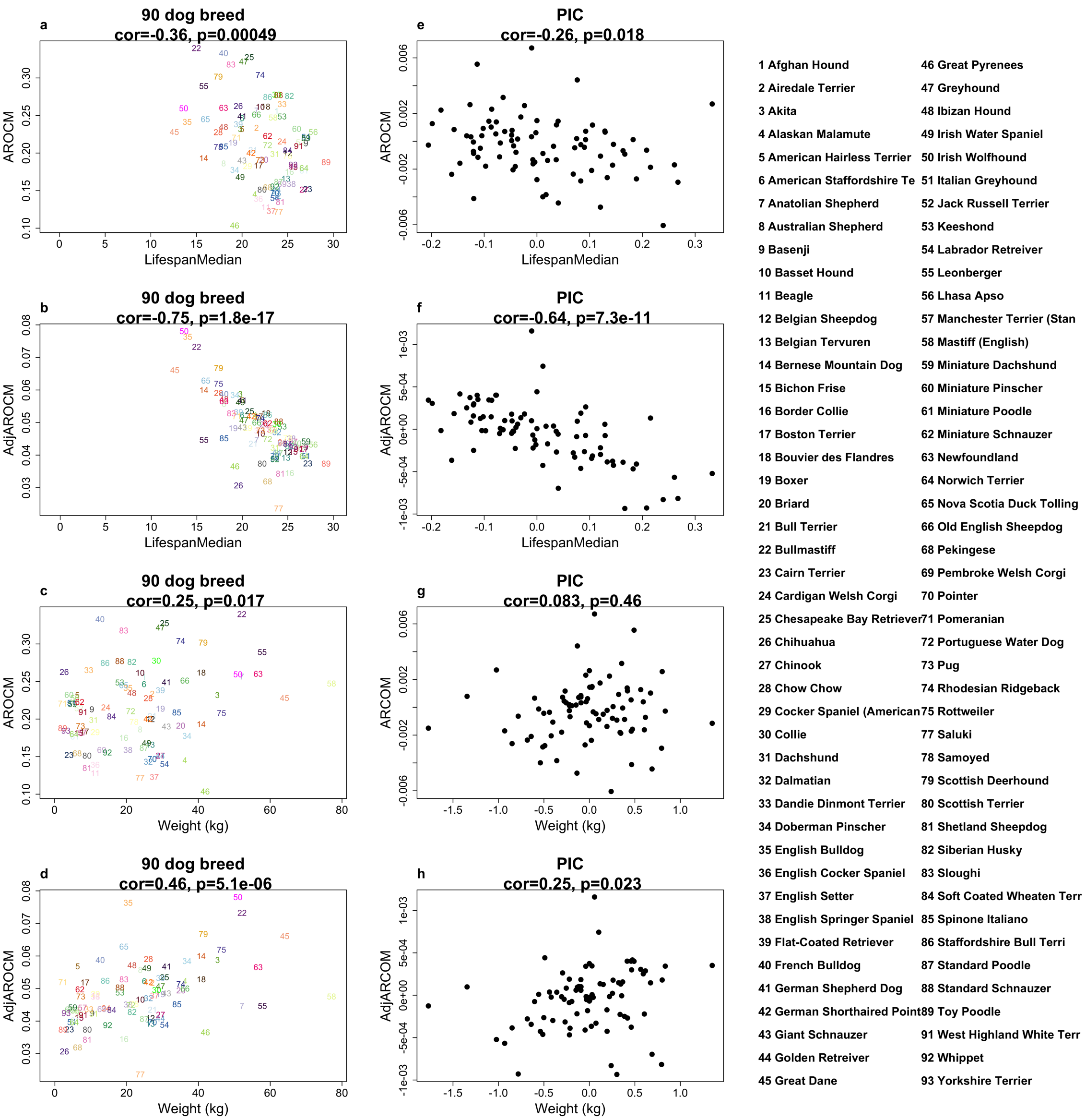

Supplementary Fig. 12: **Phylogenetic independent contrasts of AROCM in dog breeds.** Dog breed phylogenetic relationships were established as per [4]. The left panels **(a,b,c,d)** illustrate original variable relationships between N=90 dog breeds, while the right panels **(e,f,g,h)** present related results for phylogenetic independent contrasts (PICs, [2]). Accounting for these phylogenetic relationships via phylogenetic regression, both AROCM and Adj.AROCM retain significant lifespan correlations (e,f). Titles of the scatter plots report Pearson correlation coefficients and associated nominal (uncorrected) two sided Student T test p-values.

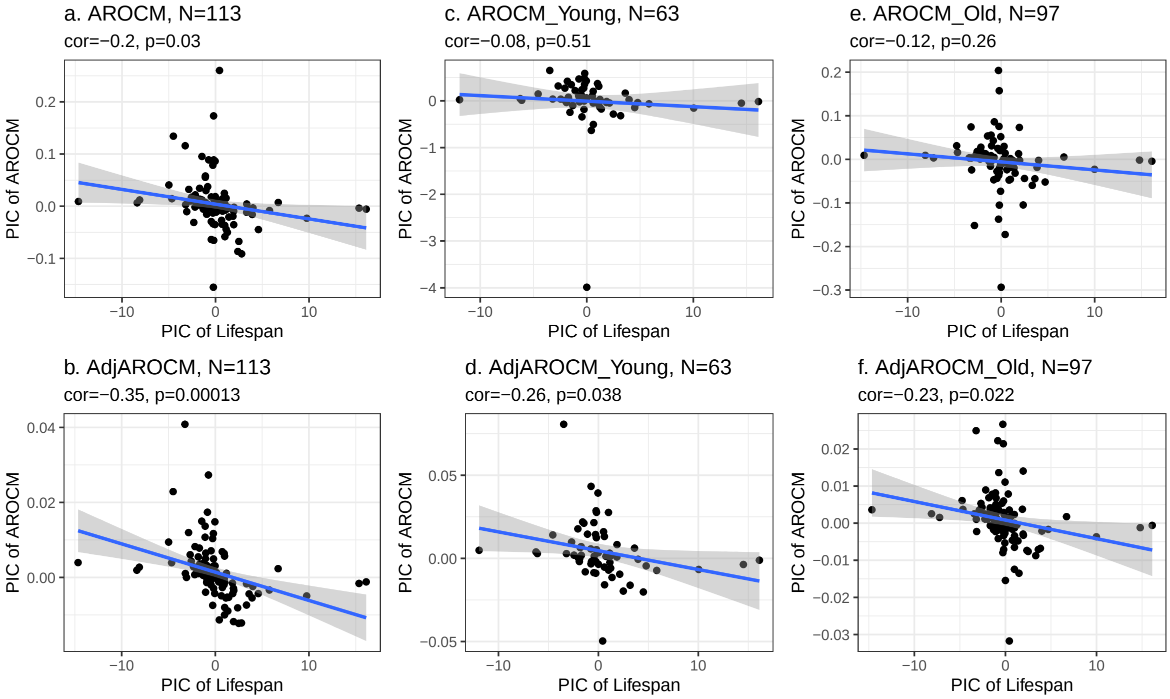

Supplementary Fig. 13: **Phylogenetic independent contrasts of AROCM in mammalian species.** The columns represent various age ranges: **a,b)** AROCM for all ages, **c,d)** young AROCM$[0, 0.1L]$, and **e,f)** old AROCM. The first and second row panels (a,c,e; b,d,f) show results for unadjusted and adjusted AROCM, respectively. Despite considering the phylogenetic links between mammalian species, the three adjusted AROCMs maintain significant lifespan associations. N denotes the sample size. The phylogenetic relationships between species come from TimeTree [5]. Titles of the scatter plots report Pearson correlation coefficients and associated nominal (uncorrected) two sided Student T test p-values.

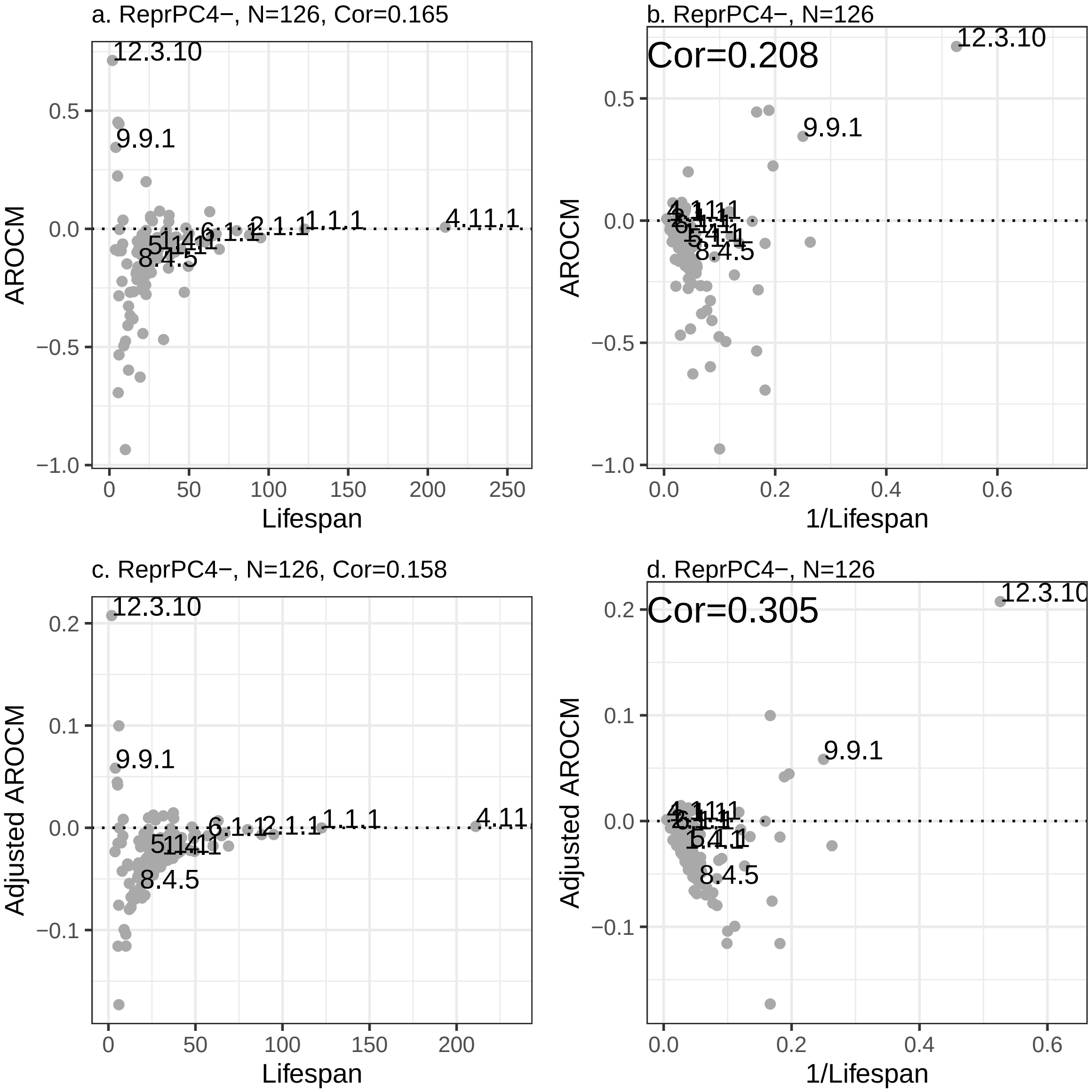

Supplementary Fig. 14: **Low correlation between AROCM and maximum lifespan in some chromatin states.** Optimal adjustment power chosen to be 0.85 for ReprPC4- in mammalian strata. **a.** AROCM vs. Lifespan; **b.** AROCM vs. 1/Lifespan; **c.** Adjusted AROCM vs. Lifespan; **d.** Adjusted AROCM vs. 1/Lifespan. Titles of the scatter plots report Pearson correlation coefficients. N denotes the sample size. This figure highlights the importance of carefully selecting which CpGs to use for defining the AROCM.

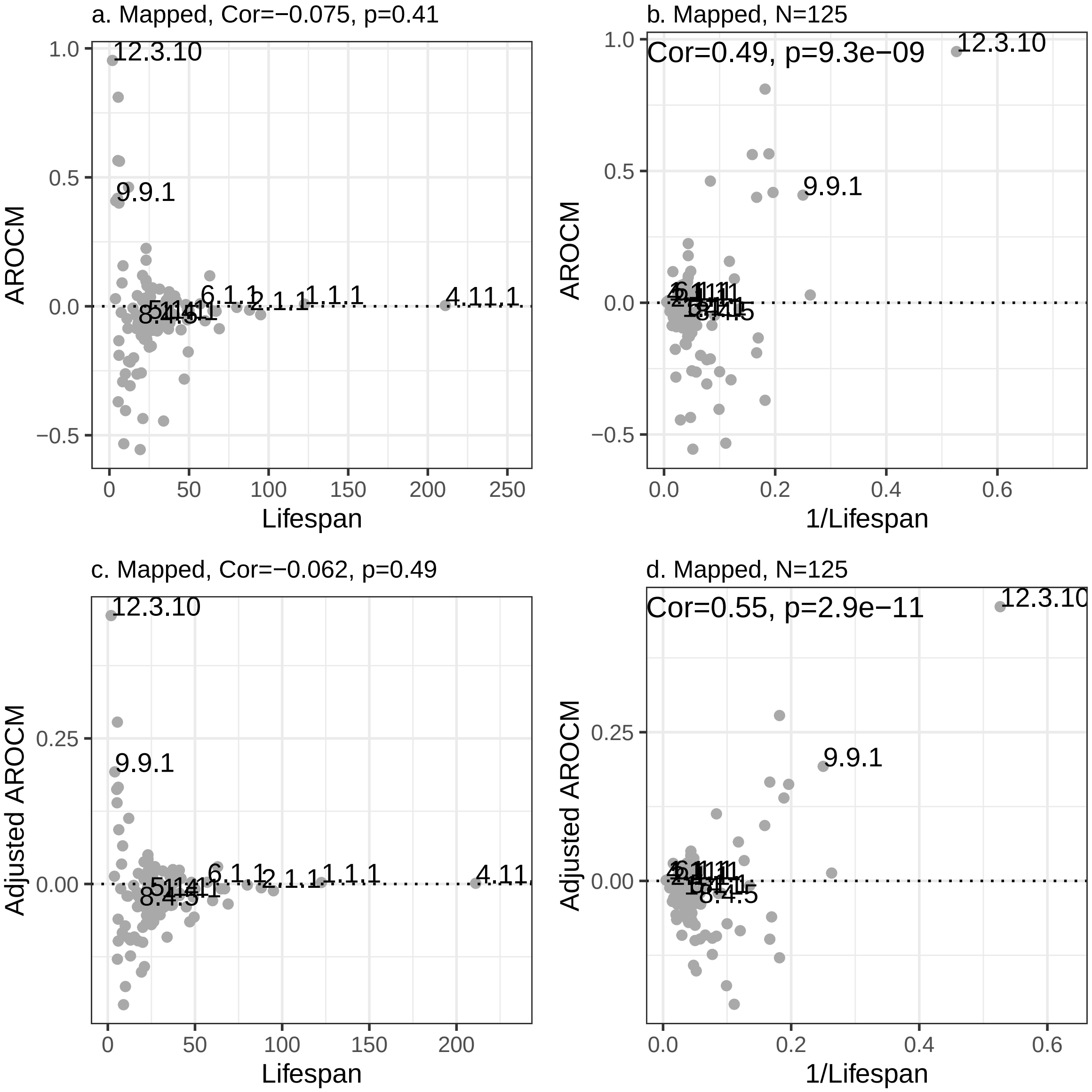

Supplementary Fig. 15: **Low correlation between AROCM and Maximum Lifespan in the 8970 CpG sites mapped to Eutherians and Marsupials.** Sample size $N = 125$. Optimal power chosen to be 0.5 for Adj.AROCM. **a.** AROCM vs. Lifespan; **b.** AROCM vs. 1/Lifespan; **c.** Adjusted AROCM vs. Lifespan; **d.** Adjusted AROCM vs. 1/Lifespan. Titles of the scatter plots report Pearson correlation coefficients. N denotes the sample size. This figure highlights the importance of carefully selecting which CpGs to use for defining the AROCM.

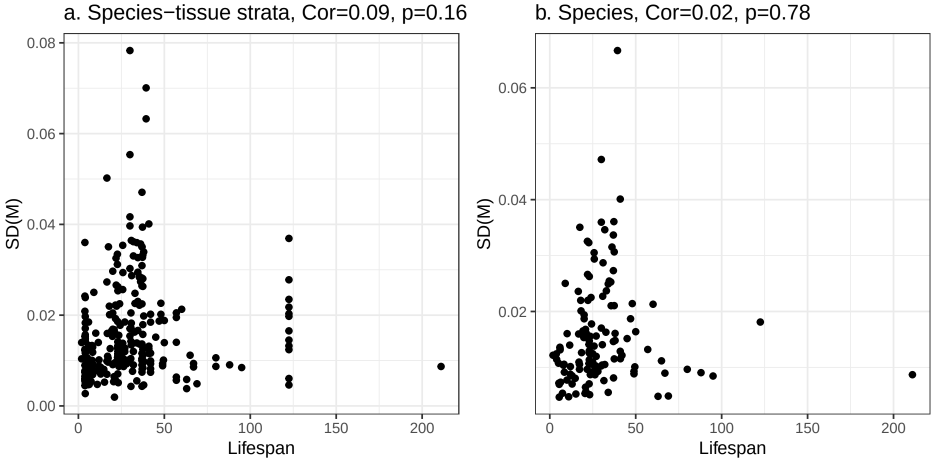

Supplementary Fig. 16: SD (M) **versus maximum lifespan in mammalian data. a.** Species-tissue strata (N=229); **b.** Species (N=125). There is no apparent dependence between the empirical standard deviation of the mean methylation and lifespan. Titles of the scatter plots report Pearson correlation coefficients and associated nominal (uncorrected) two sided Student T test p-values. N denotes the sample size.

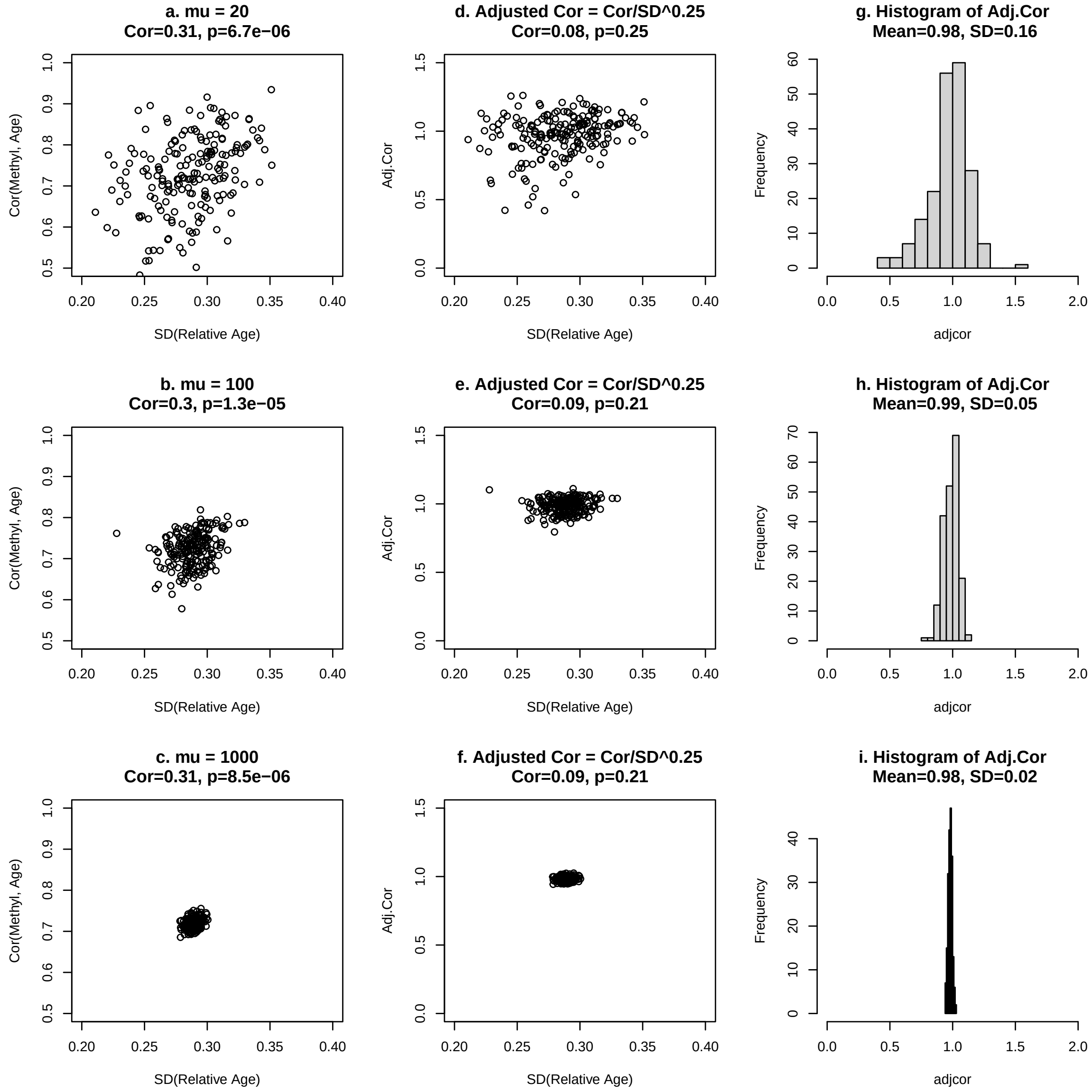

Supplementary Fig. 17: **Simulation Studies: Rationale for Adjusted Correlation Definition. Panels (a,b,c)** show age correlation cor(M,A) (y-axis) against the standard deviation of relative age (x-axis), revealing a positive Pearson correlation ($Cor \approx 0.3$) due to stratum size variation. **Panels (d,e,f)** depict Adjusted age correlation (y-axis) against the standard deviation of relative age (x-axis), where the diminished, non-significant Pearson correlation ($Cor \approx 0.09$) demonstrates robustness against variable animal counts. Titles of the scatter plots report Pearson correlation coefficients and associated nominal (uncorrected) two sided Student T test p-values. **Panels (g,h,i)** show histograms of adjusted age correlations, indicating the adjusted correlation's mean value is near 1, and its standard deviation decreases with sample size. These results suggest $Adj.$Cor approaches 1 as stratum size increases. Supplementary Note 2 provides additional details.

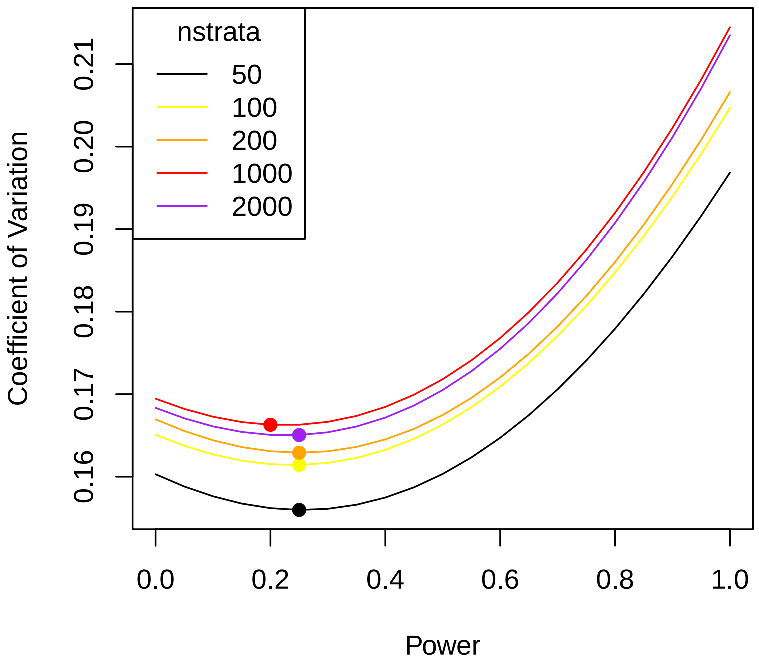

Supplementary Fig. 18: **Simulation studies to justify the definition of an adjusted correlation.** Coefficient of Variation (CV) of Adjusted Correlations as a function of different values of the adjustment power p. The CV displays a U-shape when the power increases, hence a minimum is achievable. The optimal adjustment power is achieved at 0.25 for most cases.

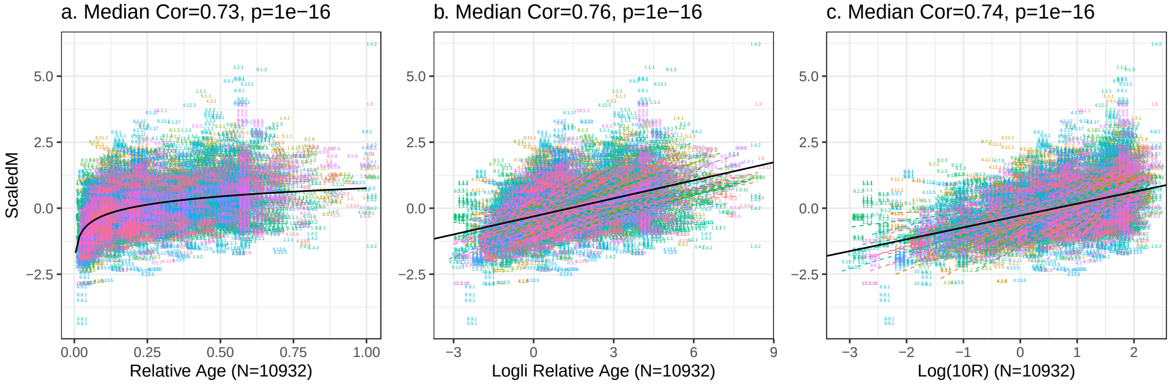

Supplementary Fig. 19: **Different relative age transformations versus ScaledM (scaled mean methylation).** Median Pearson correlation across all species is reported in the panel title as a measure of strength of association between ScaledM and (transformed) relative age. **(a)** Relative age without transformation; **(b)** Log-linear transformation of the relative age; **(c)** Log transformation of the relative age. The title reports the upper limit of a nominal two sided Student T test p-value $p < E - 16$. N denotes the sample size.

Supplementary Note 1: Data from the Mammalian Methylation Consortium

We utilized publicly available data generated by the Mammalian Methylation Consortium as described in [3]. Below, we detail the species involved, institutions, and relevant ethics protocol numbers.

- **General Mammalian Samples:** Approved by the Animal Welfare and Ethics Review Board University of Rochester Committee on Animal Resources (UCAR). Protocol #: 101939 / UCAR-2017-033.
- **Human Samples:** Approved by the University of California Los Angeles (IRB #: 15-001454, 16-000471, 18-000315, 16-002028) and the Oxford Research Ethics Committee, UK (Reference #: 10/H0605/1).
- **Yellow-bellied Marmots:** Approved by UCLA Institutional Animal Care and Use protocol (#2001-191-01, renewed annually) and with permission from Colorado Parks and Wildlife (TR917, renewed annually).
- **Zebras (Plains Zebra):** Approved by the Research Safety and Animal Welfare Administration, University of California Los Angeles: ARC #2009-090-31 (originally approved in 2009).
- **Voles:** Approved by the Institutional Animal Care and Use Committee (IACUC) of Cornell University (protocol #2013-0102) following NIH guidelines.
- **Deer Mice:** Managed by the Peromyscus Genetic Stock Center, University of South Carolina. Approved by the IACUC of the University of South Carolina (Protocol #2356-101506-042720).
- **Horses:** Approved by the UC Davis IACUC (Protocols #19037, #20751, and #21455).
- **Naked Mole Rat:** Approved by the University of Rochester Committee on Animal Resources (protocol #2009-054).
- **Beluga Whales:** Authorized under NMFS Research Permit 932-1905-00/MA-009526 and MMPA Permit #20465 issued by the National Marine Fisheries Service (NOAA).
- **Bowhead Whale:** Authorized by Fisheries and Oceans Canada (DFO) LFSP S-19/20-1007-NU and Animal Care approval (AUP) FWI-ACC-2019-14.
- **Killer Whale:** Permitted under NMFS General Authorization No. 781-1725 and scientific research permits (781-1824-01, 16163, 532-1822-00, 532-1822, 10045, 18786-03, 545-1488, 545-1761, 15616).
- **Humpback Whales:** Approved under various permits including NMFS permits (21485, 16325, 20465, 14245, 633-1483, 633-1778, 932-1905), the Canadian Department of Fisheries and Oceans, and IACUC #NWAK-18-02.
- **Cats:** Approved by the Clinical Research Ethical Review Board of the Royal Veterinary College (URN: 2019 1947-2).
- **Elephants:** Authorized by participating zoos with additional IACUC approval (#18-29) at the National Zoological Park (Smithsonian's National Zoo) and endorsement from the Elephant Taxon Advisory Group and Species Survival Plan.
- **Rats:** Approved by the Institutional Animal Ethics Committee of SVKM's NMIMS University, Mumbai (approval no. CPCSEA/IAEC/P-6/2018) adhering to CPCSEA Government of India guidelines.
- **Dogs:** Approved by the Animal Care and Use Committee of the National Human Genome Research Institute (NHGRI) at the NIH (protocol #8329254).
- **Bats:** Approved by the University of Maryland IACUC (protocol FR-APR-18-16).
- **Cattle:** Approved by the University of Nebraska IACUC (approval #1560).

- **Mice:** Approved by the University of Texas Southwestern Medical Center (APN 2015-100925 renewed every 3 years).
- **Apodemus Mice:** Approved by the University of Edinburgh Ethical Review Committee (UK Home Office Project License PP4913586).
- **Spiny Mouse:** Approved by the University of Kentucky (protocol #2019-3254).
- **Shrews and Other Small Species:** Approved by The Ohio State University IACUC (protocol #2017A00000036).

More details are provided below to make the material self-contained and more accessible for readers. We provide a list of the species and the relevant ethical approvals.

Primates

Human Ethics

This research complied with all relevant ethical regulations overseen by seven ethics review boards. The human skin samples were acquired with informed consent prior to the collection of human skin samples approved by the Oxford Research Ethics Committee in the UK; reference 10/H0605/1. Participants were not compensated. The secondary use of other de-identified/coded human tissue samples (blood, postmortem tissues) is not interpreted as human subjects research under U.S. Department of Health & Human Services 45 CFR 46. Therefore, the need to obtain written, informed consent from human study participants was waived (secondary use of de-identified tissues). Human samples were covered by University of California Los Angeles IRB#18-000315. All procedures related to non-human primates were approved by different committees: baboons (UTHSCSA Animal Care and Use Committee), strepsirrhini (Duke Institutional Animal Care and Use Committee and the DLC Research Committee), rhesus macaques (Animal Care and Use Committee of the NIA Intramural Program), vervet monkey (UCLA and VA Institutional Animal Care and Use Committees), marmosets (IACUC of UTHSA).

Baboon Care and Maintenance

All animals were given a full veterinary examination prior to recruitment to the study, and no obvious cause of ill health or pathology was observed. The animals were housed in group cages at the Southwest National Primate Research Center, at Texas Biomedical Research Institute (TBRI), in San Antonio, Texas, in mixed-sex groups of up to 16. The remaining 4 females were housed in individual cages at the UT Health Sciences Center San Antonio (UTHSCSA). Twenty-eight females and the ten males were fed ad libitum Purina Monkey Diet 5038 (12% energy from fat, 0.29% from glucose, and 0.32% from fructose and metabolizable energy content of 3.07 kcal/g protein; Purina LabDiets, St Louis, MO, USA) (CTR). Water was continuously available to all animals. Animal health status was recorded daily.

Necropsy

None of the animals were euthanized for this project. Rather, we used leftover frozen tissue samples that had previously been collected as part of other projects. Necropsies were performed by either a qualified, experienced veterinarian or M.D. investigator. At TRBI, baboons were pre-medicated with ketamine hydrochloride (10 mg/kg IM) and anesthetized using isoflurane (2%), resulting in general anesthesia. Baboons were exsanguinated while under general anesthesia as approved by the American Veterinary Medical Association. At UTHSCSA, four animals were euthanized using Pentobarbital at 390 mg/ml (Fatal-Plus Solution, Vortech, Dearborn, MI, USA). Following cardiac asystole, respiratory failure, and a lack of reflexive response to both skin pinch and eye touch stimulation, tissues (adipose, cerebellum, cerebral cortex, muscle, heart, liver) were rapidly dissected and immediately frozen in liquid nitrogen.

Fetal Tissue Studies

For the studies in which fetal tissue was obtained, all animals were housed in 20-foot × 20-foot × 15-foot metal and concrete group cages at the Texas Biomedical Research Institute. Experimental animals were obtained from appropriate groups of 16 healthy female baboons of similar pre-study body weights (10–15 kg) and morphometric features. The potential day of conception was determined based on the day of ovulation and changes in sex skin color and pregnancy was confirmed at 30 days post ovulation by using ultrasonography. All procedures were approved by the University of Texas Health Science Center and Texas Biomedical Research Institute internal animal care and use committees and performed in the Association for Assessment and Accreditation of Laboratory Animal Care–approved facilities. Prior to Cesarean section, baboons were premedicated with ketamine hydrochloride (10 mg/kg, IM). Following tracheal intubation, isoflurane (2%, 2L/min, by inhalation) was used to maintain an appropriate plane of anesthesia throughout the surgery. A cesarean section was performed at gestational day 165 (0.9 of gestation) using standard sterile techniques. Following hysterotomy, the umbilical cord was identified and used for fetal exsanguination with both maternal and fetal baboons under general anesthesia as approved by the American Veterinary Medical Association Panel on Euthanasia. Postoperatively, mothers were placed in individual cages and watched until they were upright under their own power. Maternal analgesia was administered for 3 days (buprenorphine hydrochloride injection; Hospira, Inc., Lake Forest, IL, USA; 0.015 mg/kg/day) post-operatively or longer if indicated. They were returned to their group cage two weeks postoperatively. Animals were individually fed to enable precise regulation of intake either between 7:00 am and 9:00 am or 11:00 am and 1:00 pm. Water was continuously available in each feeding cage (Lixit, Napa, California), and the animals were fed Purina Monkey Diet 5038 (Purina, St Louis, Missouri). For this study, we selected samples representing the entire primate lifespan, from neonate to old age.

Strepsirrhine Primates

Strepsirrhini is a suborder of primates that includes the lemuriform primates, which consist of the lemurs of Madagascar, pottos and galagos from Africa, and the lorises from Southeast Asia. Lemuroids and lorisoids together form the more ancestral sister clade to all other living primates. As such, they lend unparalleled power to any comparative study within the primate clade. For this study, we selected a total of 91 samples from individuals representing 26 strepsirrhine species, in most cases, the entire lifespan, from immature (infant or juvenile) to senile stages: 68 samples from peripheral blood, 23 samples from skin. The strepsirrhine primates (suborders Lemuriformes and Lorisiformes) used in this study were from the Duke Lemur Center (DLC) in Durham, NC (USA). The Duke Lemur Center is certified by both the Association for Assessment and Accreditation of Laboratory Animal Care and the American Zoological Association. The animal handling and sample collection procedures in this study were performed by a veterinarian after review and approval by the Duke Institutional Animal Care and Use Committee and the DLC Research Committee. Both housing and sample collection met or exceeded all standards of the Public Health Service's "Policy on the Humane Care and Use of Laboratory Animals". The lemurs are housed in comparable social and housing conditions, habituated to human presence, and individually identifiable. The DLC also maintains a large collection of banked tissues, deriving from routine veterinary procedures and necropsies, amassed over the Center's 55-year history. Detailed records of life and medical history, reproduction, and social-group membership are digitally maintained. Peripheral blood was collected through venipuncture with standard procedures, either during a routine veterinary procedure or at the time of necropsy. Skin tissues were collected during necropsies. Whole blood was preserved in either EDTA or Lithium Heparin and stored at -80°C. Skin tissues were either frozen directly at -80°C or were first flash frozen and then stored at -80°C.

We profiled the following species: *Cheirogaleus medius* (Fat-tailed dwarf lemur), *Daubentonia madagascariensis* (Aye-aye), *Eulemur albifrons* (White-headed lemur), *Eulemur collaris* (Collared brown lemur), *Eulemur coronatus* (Crowned lemur), *Eulemur flavifrons* (Blue-eyed black lemur), *Eulemur fulvus* (Brown lemur), *Eulemur macaco* (Black lemur), *Eulemur mongoz* (Mongoose lemur), *Eulemur rubriventer* (Red-bellied lemur), *Eulemur rufus* (Red-fronted lemur), *Eulemur sanfordi* (Sanford's brown lemur), *Galago moholi* (South African galago), *Hapalemur griseus* (Bamboo lemur), *Lemur catta* (Ring-tailed lemur), *Loris tardigradus* (Slender loris), *Microcebus murinus* (Gray mouse lemur), *Mirza zaza* (Northern giant mouse lemur), *Nycticebus coucang* (Slow loris), *Otolemur crassicaudatus*

(Greater galago), *Perodicticus potto* (Potto), *Propithecus coquereli* (Coquerel's sifaka), *Propithecus verreauxi* (Verreaux's sifaka), *Varecia rubra* (Red ruffed lemur), *Varecia variegata* (Black-and-white ruffed lemur), *Varecia variegata* (Black-and-white ruffed lemur).

Rodents

We generated methylation data on a large number of rodent tissues from 48 species including *Spalax* species that have a maximum lifespan of 21 years and a total of 91 rodent species [6].

Rodents are the largest order of mammals, comprising more than 40% of mammalian species and spanning a wide range of body sizes, lifespans, and ecologies. Among mammals, they are second only to humans as research models and the primary focus for much research on aging. This broad representation of the rodent clade includes common models (mice, rats) and a large selection of wild species with highly varied life history traits and ecologies. Our dataset includes a range of ages from immature (infant or juvenile) to senile stages, with well-documented histories in most cases. Rodent species used in this study were sampled in both laboratory and field settings, and in all cases were treated according to approved ethical standards.

Necropsy and Sample Collection

Standard procedures were used for necropsy and sample collection across all rodent species in the dataset. Animals were euthanized using carbon dioxide asphyxiation or overdose of pentobarbital, following the American Veterinary Medical Association Guidelines for the Euthanasia of Animals. Tissues (liver, muscle, heart, brain) were rapidly dissected and either flash frozen in liquid nitrogen or preserved in RNAlater and stored at -80°C.

Bats

We previously generated methylation data from a large number of bat tissues from 48 species [8]. For this study, we selected samples representing the entire bat lifespan, from neonate to old age.

Sample Collection

Bats were sampled in the field following standard ethical guidelines for capture, handling, and sampling. Field protocols were approved by relevant institutional animal care and use committees, and all necessary permits for capture and sample collection were obtained from local authorities. Bats were captured using mist nets or harp traps, and tissues (wing punch, liver, muscle, heart, brain) were collected under anesthesia (isoflurane or sevoflurane). Following sample collection, bats were either released at the site of capture or euthanized if required by the study design. Tissues were preserved in RNAlater or flash frozen in liquid nitrogen and stored at -80°C.

Mouse Lemurs

Mouse lemurs (*Microcebus murinus*) were housed at the Brunoy colony (UMR 7179 CNRS/MNHN) in France, under standard conditions of temperature (24-26°C), humidity (55%), and photoperiod (12h:12h). Animals were fed ad libitum with fresh fruits, vegetables, and a homemade protein-rich mixture. Water was provided ad libitum. Euthanasia was performed by intraperitoneal injection of pentobarbital (200 mg/kg) following sedation with ketamine (10 mg/kg). Tissues were collected immediately after euthanasia and stored at -80°C.

Supplementary Note 2: Simulation of AROCM

The fact that the number of animals per species and tissue stratum varies greatly affects both the correlation coefficients and standard deviations of different measures of interest within each stratum, as we will show using simulation studies.

Here we simulate data sets comprised of different species and tissue strata with varying sample sizes per stratum. Specifically, we use simulation studies to demonstrate that one can observe the following results:

- For stratum s ($s = 1, ..., S$), define the Pearson correlation $Cor(ScaledMeth^{(s)}, \mathbf{R}^{(s)})$ and the standard deviation of relative age $\text{SD}\left(\mathbf{R}^{(s)}\right)$. We find that these two quantities are correlated due to the different number of animals per stratum n_s.

- Adjusted Correlation, the ratio with a suitable power (e.g. $p = 0.25$),

  $$Adj.Cor(p) = Cor(ScaledM^{(s)}, \mathbf{R}^{(s)}) / \text{SD}\left(\mathbf{R}^{(s)}\right)^p,$$

  is less dependent on the standard deviation $\text{SD}\left(\mathbf{R}^{(s)}\right)$ than the correlation $Cor(ScaledM^{(s)}, \mathbf{R}^{(s)})$.

To demonstrate these results, we simulated different scenarios of data sets with $S = 200$, to mimic our real data. Each simulated stratum represents a different species whose maximum lifespan was randomly chosen to lie between 2 years (e.g. a shrew) and 250 years (e.g. long lived whales). We assumed that on a log scale, the species lifespan followed a uniform distribution, i.e., $\log L^{(s)} \sim Unif(\log 2, \log 250)$. The number of animals per stratum, n_s, was randomly sampled from a Poisson distribution whose mean value was set to μ. We explored 3 different values of $\mu = 20, 100, 1000$. In other words, $\mu = 1000$ would result in an ideal data set comprised of 1000 animals per species on average. The chronological age of each animal was randomly chosen from a uniform distribution between 0 and maximum lifespan, $A_i^{(s)} \sim Unif(0, L^{(s)})$. For each animal, the relative age was defined as the ratio between age and maximum lifespan. The mean methylation level (Methyl) was simulated to be a linear function of Relative Age, $M_i^{(s)} = R_i^{(s)} + e_i^{(s)}$, where $e_i^{(s)} \sim N(0, 0.5^2)$. For samples within each stratum, we scaled the mean methylation levels so that it would have a mean of zero and a variance of 1, i.e. $ScaledM_i^{(s)} = \frac{M_i^{(s)} - Mean(M_i^{(s)})}{\text{SD}\left(M_i^{(s)}\right)}$. Note that scaling does not change the correlation between mean methylation and age.

Within each stratum, we calculated the ratio $Adj.Cor(p) = Cor(ScaledM, \mathbf{R}) / \text{SD}\left(\mathbf{R}\right)^p$, where p is the tuning parameter power. Next, we studied the mean value, standard deviation, and the distribution of this ratio across all strata, as well as its relationship with $\text{SD}\left(\mathbf{R}^{(s)}\right)$ (Figure **Supplementary Fig. 17**). Interestingly, the correlation between $\text{Cor}(ScaledMethyl, \mathbf{R})$ (y-axis) and $\text{SD}\left(\mathbf{R}\right)$ (x-axis) remains around 0.3, independent of the average sample size per stratum (Figure **Supplementary Fig. 17** a-c), while the variances of these quantities strongly depend on the average sample size. However, the dependency between the Adjusted Correlation and $\text{SD}\left(\mathbf{R}\right)$ is much weaker and not significant (Figure **Supplementary Fig. 17** d-f). The mean of Adjusted Correlation remains the same when the average sample size increases (Figure **Supplementary Fig. 17** g-i).

Next, we investigate the power term used to define the Adjusted Correlation,

$$\begin{aligned} Adj.AROCM =& AROCM * \text{SD}\left(\mathbf{R}\right)^{1-p} \\ =& \frac{\text{Cor}(ScaledM, \mathbf{R})}{\text{SD}\left(\mathbf{R}\right)^p} \frac{1}{Lifespan}. \end{aligned} \tag{1}$$

The goal is to optimize the ratio $Adj.\text{Cor}(p) = \text{Cor}(ScaledM, \mathbf{R}) / \text{SD}\left(\mathbf{R}\right)^p$ that whose Coefficient of Variation is minimized for certain power value, $CV(p) = \text{SD}\left(Ratio(p)\right) / Mean(Ratio(p))$.

We simulated different scenarios with $S = 50, 100, 200, 1000$, and 2000, and checked how do the CVs change with respect to the power p in $Adj.\text{Cor}$ (Supplementary Figure **Supplementary Fig. 18**). The optimal power is achieved at 0.25 where the Coefficient of Variation is minimized.

For each scenario, we simulated 1000 replicate data ($nsim = 1000$) and averaged the results. In the simulation scenario that mimics our real data, we find that empirically the mean of the ratio approximates 1 when $p = 0.25$. Here we simulate the strata sample sizes following $\log n \sim N(\mu_0, \sigma_0^2)$, where μ_0, σ_0 are the empirical values from the real data.

Supplementary Note 3. Data posted Gene Expression Omnibus

Data can be accessed from individual data sets. Below, we list the GEO accession number (GEO), Title, and sample size (N).

GEO	Title	N
GSE174758	Methylation studies in prairie voles	144
GSE184211	Methylation studies in multiple human tissues N16	661
GSE184213	Methylation studies in multiple human tissues N36	96
GSE184215	Methylation studies in multiple human tissues N47	95
GSE184216	Methylation studies in roe deer	94
GSE184218	Methylation studies in multiple human tissues N66	92
GSE184220	Methylation studies in human and mouse placenta	47
GSE184221	Methylation studies in human blood N94 N68	384
GSE184224	Methylation studies in human cells N76	17
GSE190660	Methylation studies in the vervet monkey	240
GSE190661	Methylation studies in baboons	325
GSE190662	Methylation studies in strepsirrhini primates	91
GSE190663	Methylation studies in common marmoset	96
GSE190664	Methylation studies in rhesus macaque	283
GSE174544	Methylation studies in yellow-bellied marmots	159
GSE190665	In vivo partial reprogramming in 4F mice	168
GSE174767	Methylation studies in horses	333
GSE184222	Methylation studies in wild ass and Grevy's zebra	12
GSE184223	Methylation studies in plains zebras	118
GSE174777	Methylation studies in naked mole rats. Part 1	289
GSE174778	Methylation studies in mole rats. Part 2	94
GSE173330	Methylation clocks for age estimation in toothed whales and dolphins	545
GSE164127	Genome Methylation Predicts Age and Longevity of Bats	908
GSE147002	DNA methylation profiles from a mouse model of Huntington's disease	72
GSE147003	DNA methylation profiles from a transgenic sheep model of Huntington's disease	168
GSE147004	DNA Methylation Study of Huntington's Disease and Motor Progression in Three Species	348

Supplementary Data

Supplementary data can be found in the Supplementary Data file.

Legends of Supplementary Data

Supplementary Data 1. Characteristics of 93 Dog Breeds. This table presents various characteristics of 93 dog breeds, including the median breed lifespan, upper confidence interval limit of breed lifespan, and average breed weight (in kilograms). The source of the breed information is from the article by Horvath (2022) published in PNAS (PubMed Identifier: 35580182). Additional columns include:

- Breed Index: Identification index for each dog breed.
- Sample Size: The number of blood samples collected per dog breed.
- SD_{Age}: Standard deviation of chronological age in years.
- SD_{Rage}: Standard deviation of relative age.
- SD_{Methyl}: Standard deviation of the mean methylation levels.
- Methyl: Mean methylation of CpGs located in chromatin state BivProm2+.
- Cor(Age, Methyl): Pearson correlation coefficient between age and methylation.
- AROCM: Average rate of change in methylation in chromatin state BivProm2+.
- R2: R-squared from the linear regression of fitting the AROCM.

Supplementary Data 2. 54 Chromatin States and their characteristics. The universal chromatin states are described in [7]. Column denoted as statesPRC2 relates to subsets of CpGs within each chromatin state based on whether the CpG lies in a PRC2 binding site. The chromatin states were used in several papers from the mammalian methylation consortium e.g. [6]. Other columns include the number of CpGs in the chromatin state (Freq), Number of CpGs with a positive slope/correlation with age.

- Cor1/slope denotes the Pearson correlation between the mean methylation and 1/slope.
- Cor1/Lifespan denotes the Pearson correlation between mean methylation and 1/Lifespan.
- CorLog denotes the Pearson correlation between log of mean methylation and log Lifespan.
- CorSpearman denotes the Spearman correlation between mean methylation and Lifespan.

Supplementary Data 3. Species-tissue strata characteristics: This table summarizes the characteristics of different species-tissue combinations, including the species name (both Latin and common names), the Mammalian Order Number as defined by [6], sample size, age range (expressed in units of relative age), and AROCMs (slopes) across all chromatin states. The species characteristics (such as maximum lifespan, gestation time, etc.) are sourced from an adapted version of the AnAge database, as described by [6]. The original AnAge data base (created by Joao Pedro de Magaelhaes) can be found here: https://genomics.senescence.info/species/index.html. Starting from column Q, there are 13 AROCMs calculated per chromatin state with column names

- "statenameYoungX", AROCM based on age range $(0, X * Lifespan)$,
- "statenameOldY", AROCM based on age range $(Y * ASM, Lifespan)$.

Supplementary Data 4. AROCMs and Adjusted AROCMs by Species (alphabetic order from row 6) and lemur families (rows 1 to 5) for BivProm2+. These are based on age range (0, Lifespan).

Supplementary Data 5. List of CpGs Located in the Chromatin State BivProm2+: The mean methylation level of these CpGs is used in the calculation of the AROCM. These CpGs are part of the mammalian methylation array (HorvathMammalMethylChip40) as described by [1]. Genome coordinates in hundreds of species can be found in the annotation files from the data availability statements in [1] and [3].

References

[1] A. Arneson, A. Haghani, M. J. Thompson, M. Pellegrini, S. B. Kwon, H. Vu, E. Maciejewski, M. Yao, C. Z. Li, A. T. Lu, et al. A mammalian methylation array for profiling methylation levels at conserved sequences. *Nature communications*, 13(1):1–13, 2022.

[2] J. Felsenstein. Phylogenies and the comparative method. *The American Naturalist*, 125(1):1–15, 1985.

[3] A. Haghani, C. Z. Li, T. R. Robeck, J. Zhang, A. T. Lu, J. Ablaeva, V. A. Acosta-Rodríguez, D. M. Adams, A. N. Alagaili, J. Almunia, et al. Dna methylation networks underlying mammalian traits. *Science*, 381(6658):eabq5693, 2023.

[4] S. Horvath, A. T. Lu, A. Haghani, J. A. Zoller, C. Z. Li, A. R. Lim, R. T. Brooke, K. Raj, A. Serres-Armero, D. L. Dreger, et al. Dna methylation clocks for dogs and humans. *Proceedings of the National Academy of Sciences*, 119(21):e2120887119, 2022.

[5] S. Kumar, G. Stecher, M. Suleski, and S. B. Hedges. Timetree: a resource for timelines, timetrees, and divergence times. *Molecular biology and evolution*, 34(7):1812–1819, 2017.

[6] A. T. Lu, Z. Fei, A. Haghani, T. R. Robeck, J. Zoller, C. Li, R. Lowe, Q. Yan, J. Zhang, H. Vu, et al. Universal dna methylation age across mammalian tissues. *Nature aging*, 3(9):1144–1166, 2023.

[7] H. Vu and J. Ernst. Universal annotation of the human genome through integration of over a thousand epigenomic datasets. *Genome biology*, 23(1):1–37, 2022.

[8] G. S. Wilkinson, D. M. Adams, A. Haghani, A. T. Lu, J. Zoller, C. E. Breeze, B. D. Arnold, H. C. Ball, G. G. Carter, L. N. Cooper, et al. Dna methylation predicts age and provides insight into exceptional longevity of bats. *Nature communications*, 12(1):1–13, 2021.
