# Supplementary material for: Fundamental equations linking methylation dynamics to maximum lifespan in mammals
Source: Nat Commun. 2024 Sep 16;15:8093. doi: 10.1038/s41467-024-51855-z (PMC11405513; doi:10.1038/s41467-024-51855-z)
Supplement: Supplementary file 3 — Description of Additional Supplementary Files [file 41467_2024_51855_MOESM3_ESM.pdf]

Description of Additional Supplementary Files

File Name: Supplementary Data 1
Description: Characteristics of 93 Dog Breeds: This table includes various characteristics of 93 dog breeds, including breed index, sample size, average lifespan, standard deviation of age (SD_Age), and mean methylation levels (SD_Methyl).

File Name: Supplementary Data 2
Description: 54 Chromatin States and their characteristics.

File Name: Supplementary Data 3
Description: Species-tissue strata Characteristics: This table summarizes the characteristics of different species-tissue combinations, including their indices, sample sizes, lifespans, age range, and AROCMs in all chromatin states.

File Name: Supplementary Data 4
Description: AROCMs and Adjusted AROCMs by Species (alphabetic order from row 6) and lemur families (rows 1 to 5) for BivProm2+.

File Name: Supplementary Data 5
Description: List of CpGs in the chromatin state BivProm2+.
