# Supplementary material for: Fundamental equations linking methylation dynamics to maximum lifespan in mammals
Source: Nat Commun. 2024 Sep 16;15:8093. doi: 10.1038/s41467-024-51855-z (PMC11405513; doi:10.1038/s41467-024-51855-z)
Supplement: Supplementary file 5 — Reporting Summary [file 41467_2024_51855_MOESM5_ESM.pdf]

nature portfolio

Corresponding author(s): Zhe Fei

Last updated by author(s): Aug 16, 2024

Reporting Summary

Nature Portfolio wishes to improve the reproducibility of the work that we publish. This form provides structure for consistency and transparency in reporting. For further information on Nature Portfolio policies, see our Editorial Policies and the Editorial Policy Checklist.

Statistics

For all statistical analyses, confirm that the following items are present in the figure legend, table legend, main text, or Methods section.

n/a	Confirmed	
☐	☒	The exact sample size (*n*) for each experimental group/condition, given as a discrete number and unit of measurement
☐	☒	A statement on whether measurements were taken from distinct samples or whether the same sample was measured repeatedly
☐	☒	The statistical test(s) used AND whether they are one- or two-sided<br>*Only common tests should be described solely by name; describe more complex techniques in the Methods section.*
☐	☒	A description of all covariates tested
☐	☒	A description of any assumptions or corrections, such as tests of normality and adjustment for multiple comparisons
☐	☒	A full description of the statistical parameters including central tendency (e.g. means) or other basic estimates (e.g. regression coefficient) AND variation (e.g. standard deviation) or associated estimates of uncertainty (e.g. confidence intervals)
☐	☒	For null hypothesis testing, the test statistic (e.g. F, t, r) with confidence intervals, effect sizes, degrees of freedom and P value noted<br>*Give P values as exact values whenever suitable.*
☒	☐	For Bayesian analysis, information on the choice of priors and Markov chain Monte Carlo settings
☒	☐	For hierarchical and complex designs, identification of the appropriate level for tests and full reporting of outcomes
☐	☒	Estimates of effect sizes (e.g. Cohen's d, Pearson's r), indicating how they were calculated

Our web collection on statistics for biologists contains articles on many of the points above.

Software and code

Policy information about availability of computer code

Data collection	We do not present findings from specific experiments. Rather, DNA samples were collected opportunistically from available freezer-stored materials provided by our collaborators. Data collection and analysis were not conducted blind to variables such as tissue type. Methylation measurements were taken from different animals, ensuring that no animal was measured more than once. Details can be found in Supplementary Note 1. Further, the dog blood samples are described in Horvath et al. 2022 PNAS paper "DNA methylation clocks for dogs and humans" (doi.org/10.1073/pnas.2120887119). The samples are maintained as Excel spread sheets or Rdata generated in R. Collections of Mammalian samples are described in Lu et al. 2021 paper "Universal DNA methylation age across mammalian tissues" (doi.org/10.1101/2021.01.18.426733). Mammalian samples are maintained as Excel spread sheets or Rdata generated in R.
Data analysis	R_ 4.2.0: Programming language for statistical computing<br>R_WGCNA_l.69: Weighted correlation network analysis for analysis and graphics.<br>R_ggplot2, R_gridExtra: for figures including box plots, bar plots, scatter plots, and smoothing curves.<br>R_ape: for calculating phylogenetic independent contrasts of AROCM.

For manuscripts utilizing custom algorithms or software that are central to the research but not yet described in published literature, software must be made available to editors and reviewers. We strongly encourage code deposition in a community repository (e.g. GitHub). See the Nature Portfolio guidelines for submitting code & software for further information.

Data

Policy information about availability of data

All manuscripts must include a data availability statement. This statement should provide the following information, where applicable:

- Accession codes, unique identifiers, or web links for publicly available datasets
- A description of any restrictions on data availability
- For clinical datasets or third party data, please ensure that the statement adheres to our policy

The data for all species from the Mammalian Methylation Consortium can be downloaded from Gene Expression Omnibus (GEO) using the accession number GSE223748. To facilitate comparative analyses across species, the consortium applied a single measurement platform (the mammalian methylation array, GPL28271) to n=15,216 DNA samples derived from 70 tissue types of 348 different mammalian species (331 eutherian-, 15 marsupial-, and 2 monotreme species). The 11,754 samples used for training our universal clocks are part of the samples, which are available for age information.
The individual-level data from the Mammalian Methylation Consortium can be accessed from several online locations. All data from the Mammalian Methylation Consortium are posted on Gene Expression Omnibus (complete dataset, GSE223748 [https://www.ncbi.nlm.nih.gov/geo/query/acc.cgi?acc=GSE223748] \citep{lu2023universal,haghani2023}). Additional details can be found in Supplementary Note 3. The mammalian methylation array is available through the non-profit Epigenetic Clock Development Foundation (https://clockfoundation.org/).
Numerical results generated in this study are provided in the Supplementary Information/Source Data file.
Subset of the data can be downloaded from GEO using the following accession numbers: GSE174758, GSE184211, GSE184213, GSE184215,GSE184216,GSE184218,GSE184220,GSE184221,GSE184224,GSE190660,GSE190661,GSE190662,GSE190663,GSE190664,GSE174544,GSE190665, GSE174767, GSE184222, GSE184223, GSE174777, GSE174778, GSE173330, GSE164127, GSE147002, GSE147003, GSE147004. The mammalian methylation array is available through the non-profit Epigenetic Clock Development Foundation (https://clockfoundation.org/).

Research involving human participants, their data, or biological material

Policy information about studies with human participants or human data. See also policy information about sex, gender (identity/presentation), and sexual orientation and race, ethnicity and racism.

Reporting on sex and gender	Not applicable. Both sexes, roughly half and half.
Reporting on race, ethnicity, or other socially relevant groupings	Not applicable.
Population characteristics	Not applicable.
Recruitment	Not applicable.
Ethics oversight	Not applicable. We used publicly available data. The secondary use of the other de-identified/coded human tissue samples (blood, postmortem tissues) is not interpreted as human subjects research under U.S. Department of Health and Human Services 45 CFR 46. Therefore, the need to obtain written, informed consent from human study participants was waived (secondary use of de-identified tissues). Human samples were covered by University of California Los Angeles IRB number 18-000315.

Note that full information on the approval of the study protocol must also be provided in the manuscript.

Field-specific reporting

Please select the one below that is the best fit for your research. If you are not sure, read the appropriate sections before making your selection.

☐ Life sciences ☐ Behavioural & social sciences ☒ Ecological, evolutionary & environmental sciences

For a reference copy of the document with all sections, see nature.com/documents/nr-reporting-summary-flat.pdf

Ecological, evolutionary & environmental sciences study design

All studies must disclose on these points even when the disclosure is negative.

Study description	DNA samples were collected opportunistically from available freezer-stored materials provided by our collaborators. Observational data based on existing samples stored in freezers. We generated 10,932 methylation arrays from over 57 tissue-types derived from 133 mammalian species. We aimed to profile animals from the entire age range: from very young to very old. Roughly uniform distribution. We only analyzed tissues from animals whose ages were known with 90% confidence.
Research sample	I. We employed a custom methylation array (HorvathMammalMethylChip40) that profiles methylation levels of 36k CpGs with flanking DNA sequences that are highly-conserved across the mammalian class.<br>II. We obtained such profiles from 11,754 samples from 59 tissue types, derived from 185 mammalian species, representing 19

taxonomic orders and ranging in age from prenatal to 139 years old (bowhead whale).
III. The tissue samples are described in the Supplement and related citations as listed in Supplementary Information, Note 1.
IV. To enhance the reproducibility of our findings we include our updated version of the animal age (anAge) database, which is reported in the supplementary data.
V. Below are the list of ipapers that describe specific species:
1. Horvath, S. et al. Pan-primate DNA methylation clocks. bioRxiv, 2020.11.29.402891 (2021).
2. Horvath, S. et al. Epigenetic clock and methylation studies in the rhesus macaque. GeroScience (2021).
3. Jasinska, A.J. et al. Epigenetic clock and methylation studies in vervet monkeys. GeroScience (2021).
4. Horvath, S. et al. DNA methylation age analysis of rapamycin in common marmosets. GeroScience (2021).
5. Schlabritz-Loutsevitch, N.E. et al. Metabolic adjustments to moderate maternal nutrient restriction. British journal of nutrition 98, 276-284 (2007).
6. Kavitha, J.V. et al. Down-regulation of placental mTOR, insulin/IGF-I signaling, and nutrient transporters in response to maternal nutrient restriction in the baboon. FASEB journal : official publication of the Federation of American Societies for Experimental Biology 28, 1294-1305 (2014).
7. Schlabritz-Loutsevitch, N.E. et al. Development of a system for individual feeding of baboons maintained in an outdoor group social environment. Journal of Medical Primatology 33, 117-126 (2004).
8. Zehr, S.M. et al. Life history profiles for 27 strepsirrhine primate taxa generated using captive data from the Duke Lemur Center. Scientific Data 1, 140019 (2014).
9. Morgello, S. et al. The National NeuroAIDS Tissue Consortium: a new paradigm in brain banking with an emphasis on infectious disease. Neuropathol Appl Neurobiol 27, 326-35. (2001).
10. Horvath, S. et al. HIV, pathology and epigenetic age acceleration in different human tissues. Geroscience (2022).
11. Horvath, S. et al. Perinatally acquired HIV infection accelerates epigenetic aging in South African adolescents. AIDS (London, England) 32, 1465-1474 (2018).
12. Horvath, S. & Ritz, B.R. Increased epigenetic age and granulocyte counts in the blood of Parkinson's disease patients. Aging (Albany NY) 7, 1130-42 (2015).
13. Kabacik, S., Horvath, S., Cohen, H. & Raj, K. Epigenetic ageing is distinct from senescence-mediated ageing and is not prevented by telomerase expression. Aging (Albany NY) 10, 2800-2815 (2018).
14. Ross, C.N. et al. The development of a specific pathogen free (SPF) barrier colony of marmosets (Callithrix jacchus) for aging research. Aging (Albany NY) 9, 2544 (2017).
15. Sailer, L.L. et al. Pair bonding slows epigenetic aging and alters methylation in brains of prairie voles. bioRxiv, 2020.09.25.313775 (2020).
16. Ophir, A.G. Navigating Monogamy: Nonapeptide Sensitivity in a Memory Neural Circuit May Shape Social Behavior and Mating Decisions. Frontiers in Neuroscience 11(2017).
17. Horvath, S. et al. Methylation studies in Peromyscus: aging, altitude adaptation, and monogamy. GeroScience 44, 447-461 (2022).
18. Horvath, S. et al. DNA methylation aging and transcriptomic studies in horses. Nat Commun 13, 40 (2022).
19. Burns, E.N. et al. Generation of an equine biobank to be used for Functional Annotation of Animal Genomes project. Animal genetics 49, 564-570 (2018).
20. Horvath, S. et al. DNA methylation clocks tick in naked mole rats but queens age more slowly than nonbreeders. Nature Aging 2, 46-59 (2022).
21. Ke, Z., Vaidya, A., Ascher, J., Seluanov, A. & Gorbunova, V. Novel husbandry techniques support survival of naked mole rat (Heterocephalus glaber) pups. J Am Assoc Lab Anim Sci 53, 89-91 (2014).
22. Tan, L. et al. Naked Mole Rat Cells Have a Stable Epigenome that Resists iPSC Reprogramming. Stem cell reports 9, 1721-1734 (2017).
23. Sugrue, V.J. et al. Castration delays epigenetic aging and feminizes DNA methylation at androgen-regulated loci. eLife 10, e64932 (2021).
24. Schachtschneider, K.M. et al. Epigenetic clock and DNA methylation analysis of porcine models of aging and obesity. GeroScience (2021).
25. Robeck, T.R. et al. Multi-Tissue Methylation Clocks for Age and Sex Estimation in the Common Bottlenose Dolphin. Frontiers in Marine Science 8(2021).
26. Robeck, T.R. et al. Multi-species and multi-tissue methylation clocks for age estimation in toothed whales and dolphins. Commun Biol 4, 642 (2021).
27. Bors, E.K. et al. An epigenetic clock to estimate the age of living beluga whales. Evolutionary Applications (2020).
28. Raj, K. et al. Epigenetic clock and methylation studies in cats. GeroScience (2021).
29. Prado, N.A. et al. Epigenetic clock and methylation studies in elephants. Aging Cell 20, e13414 (2021).
30. Pinho, G.M. et al. Hibernation slows epigenetic ageing in yellow-bellied marmots. Nature Ecology & Evolution 6, 418-426 (2022).
31. Lemaitre, J.-F. et al. DNA methylation as a tool to explore ageing in wild roe deer populations. Molecular Ecology Resources n/a(2021).
32. Larison, B. et al. Epigenetic models developed for plains zebras predict age in domestic horses and endangered equids. Communications Biology 4, 1412 (2021).
33. Harley, E.H., Knight, M.H., Lardner, C., Wooding, B. & Gregor, M. The Quagga project: progress over 20 years of selective breeding. African Journal of Wildlife Research 39, 155-163 (2009).
34. Horvath, S. et al. Reversing age: dual species measurement of epigenetic age with a single clock. bioRxiv, 2020.05.07.082917 (2020).
35. Horvath, S. et al. Epigenetic clock and methylation studies in dogs. PNAS In Press(2022).
36. Plassais, J. et al. Whole genome sequencing of canids reveals genomic regions under selection and variants influencing morphology. Nature Communications 10, 1489 (2019).
37. Plassais, J. et al. Analysis of large versus small dogs reveals three genes on the canine X chromosome associated with body weight, muscling and back fat thickness. PLOS Genetics 13, e1006661 (2017).
38. TheAmericanKennelClub. The Complete Dog Book: 20th Edition, (Howell Book House, New York, NY, 2006).
39. Wilcox, B. & Walkowicz, C. The Atlas of Dog Breeds of the World, (T.F.H. Publications, 1995).
40. Wilkinson, G.S. et al. DNA methylation predicts age and provides insight into exceptional longevity of bats. Nature Communications 12, 1615 (2021).
41. Kordowitzki, P. et al. Epigenetic clock and methylation study of oocytes from a bovine model of reproductive aging. Aging Cell 20, e13349 (2021).
42. Mozhui, K. et al. Genetic loci and metabolic states associated with murine epigenetic aging. eLife 11, e75244 (2022).
43. Lu, A.T. et al. DNA methylation study of Huntington's disease and motor progression in patients and in animal models. Nature

Communications 11, 4529 (2020).
44. Coschigano, K. et al. Deletion, but not antagonism, of the mouse growth hormone receptor results in severely decreased body weights, insulin, and insulin-like growth factor I levels and increased life span. Endocrinology 144, 3799-3810 (2003).
45. Acosta-Rodriguez, V.A., Rijo-Ferreira, F., Green, C.B. & Takahashi, J.S. Importance of circadian timing for aging and longevity. Nature Communications 12, 2862 (2021).
46. Little, T.J. et al. Methylation-Based Age Estimation in a Wild Mouse. bioRxiv, 2020.07.16.203687 (2020).
47. Cossette, M.-L. et al. Differential methylation, epigenetic clocks, and island-mainland divergence in an insectivorous small mammal. bioRxiv, 2022.04.14.488253 (2022).
48. Horvath, S. et al. Epigenetic clock and methylation studies in marsupials: opossums, Tasmanian devils, kangaroos, and wallabies. Geroscience In Press(2022).
49. Hogg, C.J., Lee, A.V. & Hibbard, C.J. Managing a metapopulation: intensive to wild and all the places in between. in Saving the Tasmanian Devil: recovery through science based management 169-182 (CSIRO Publishing Melbourne, 2019).
50. Hogg, C. & Hockley, J. DPIPWE/ZAA husbandry guidelines for Tasmanian devil, Sarcophilus harrisii. Australia: Zoo and Aquarium Association (2013).
51. Sambrook, J. & Russell, D.W. Purification of nucleic acids by extraction with phenol: chloroform. Cold Spring Harbor Protocols 2006, pdb. prot4455 (2006).
52. Villar, D. et al. Enhancer evolution across 20 mammalian species. Cell 160, 554-66 (2015).
53. Berthelot, C., Villar, D., Horvath, J.E., Odom, D.T. & Flicek, P. Complexity and conservation of regulatory landscapes underlie evolutionary resilience of mammalian gene expression. Nat Ecol Evol 2, 152-163 (2018).
54. Roller, M. et al. LINE retrotransposons characterize mammalian tissue-specific and evolutionarily dynamic regulatory regions. Genome Biol 22, 62 (2021).
55. Yan, L. et al. OSAT: a tool for sample-to-batch allocations in genomics experiments. BMC Genomics 13, 689 (2012).
56. Seluanov, A. et al. Hypersensitivity to contact inhibition provides a clue to cancer resistance of naked mole-rat. Proceedings of the National Academy of Sciences 106, 19352-19357 (2009).
57. Seluanov, A. et al. Telomerase activity coevolves with body mass not lifespan. Aging Cell 6, 45-52 (2007).

Sampling strategy

We sampled all mammalian species for which existing tissues were available. These fresh frozen tissue samples were contributed by a large network of investigators from our Mammalian Methylation Consortium.
To guide the quality control (QC) of the study samples, we generated two variables ; the first being a variable indicating the confidence (0 to 100%) in the chronological age estimate of the sample. For example, a low confidence was assigned to samples from wild animals whose ages were estimated based on body length measurements. The epigenetic clocks were trained and evaluated in tissue samples whose confidence exceeded 90% (>=90%). The second quality control variable was an indicator variable (yes/no) that flagged technical outliers or malignant (cancer) tissue. Since we were interested in "normal" aging patterns we excluded tissues from preclinical studies surrounding anti-aging or pro-aging interventions.

Data collection

DNA for methylation profiling was extracted from the tissue samples collected from different species as described in Supplementary Information, Note 1 . After bisulfite conversion and labeling of the DNA, methylation profiles were obtained by hybridizing labeled DNA to a custom Illumina methylation array (HorvathMammalMethylChip40) and scanning with an Illumina iScan at the UCLA Neuroscience Genomics Core.

Timing and spatial scale

The tissue samples were collected over the last 30 years. The data come from many labs all over the world: US, Canada, Europe, Australia, New Zealand, South America.

Data exclusions

We excluded about 1900 samples that had insufficient DNA to provide reliable methylation values, low confidence in the chronological age estimate or unknown age. We discovered that samples with concentrations below 6 ng/µl could not be accurately scored at all sites on the array.

Reproducibility

1. We used calibration data (synthetic DNA) to evaluate the accuracy of the methylation measurements (A mammalian methylation array for profiling methylation levels at conserved sequences by A. Arneson 2021 Nat Comm)
2. We performed EWAS meta-analysis of age using Stouffer's method estimates from Metal algorithm. In addition, we verified the Stouffer's statistics in our in-house R code.
3. The universal clocks were established via elastic net regression models. To assess the accuracies of our clocks, we used 3 approaches: leave-one-fraction-out (LOFO), leave one-species-out (LOSO) cross validation, and data splitting.
In LOFO, we randomly split the entire dataset into 10 fractions each of which had the same distribution in species and tissue types. Each penalized regression model was trained in 9 fractions but evaluated in the 10th left out fraction. After circling through the 10 fractions, we arrived at LOFO predictions which were subsequently related to the actual values.
The LOSO cross validation approach trained each model on all but one species. The left out species was used a test set. The LOSO approach was used to assess how well the penalized regression models generalize to species that were not part of the training data.
To ensure unbiased estimates of accuracy, all aspects of the model fitting were only conducted in the training data in both LOFO and LOSO analysis.
4. The reported EWAS p values are significant even after using the most stringent multiple comparison correction (Bonferroni)=0.05/37K based on 37K CpGs on the mammalian array.
5. In GREAT enrichment analysis, we performed two different sensitivity analyses that were inspired by our GREAT enrichment analysis of the top 1 thousand age related CpGs (EWAS of age). The results are listed in Supplementary Info, Note 2. Our first sensitivity analysis involved a random set of 1000 CpG mammalian CpGs. Second, we evaluated the enrichment of the top 1087 most highly conserved CpGs across 158 mammalian genomes. This sensitivity analysis addresses the concern that highly conserved CpGs could have an increased chance of correlating strongly with chronological age or, conversely, non-conserved (noise) CpGs are expected to have no signal for age and will therefore not be selected in an EWAS of age.
6. In single cell AT AC-seq analysis, to confirm enrichment for the hyper methylated sites showing decrease of chromatin accessibility with age, we randomly selected 1000 sets of 17 ATAC peaks and compared the mean correlation with age of the selected regions to the 1000 sampled sets of regions.

Randomization	Not applicable since this is an observational study.
Blinding	Blinding was not relevant to our study, because this is an observational study and all available data were used

Did the study involve field work? ☐ Yes ☒ No

Reporting for specific materials, systems and methods

We require information from authors about some types of materials, experimental systems and methods used in many studies. Here, indicate whether each material, system or method listed is relevant to your study. If you are not sure if a list item applies to your research, read the appropriate section before selecting a response.

Materials & experimental systems

n/a	Involved in the study
☒	☐ Antibodies
☒	☐ Eukaryotic cell lines
☒	☐ Palaeontology and archaeology
☐	☒ Animals and other organisms
☒	☐ Clinical data
☒	☐ Dual use research of concern
☒	☐ Plants

Methods

n/a	Involved in the study
☒	☐ ChIP-seq
☒	☐ Flow cytometry
☒	☐ MRI-based neuroimaging

Animals and other research organisms

Policy information about studies involving animals; ARRIVE guidelines recommended for reporting animal research, and Sex and Gender in Research

Laboratory animals	This study leveraged existing tissue samples or data that had been collected as part of other studies. We profiled tissues from lab animals or animals kept in captivity for research. This includes mouse, rat, opossum, naked mole rat, deer mouse colonies, rhesus macaque, marmosets, vervet monkey. Companion pets: dogs, cats. Agricultural animals: horses, pigs, sheep. Details in Supplementary Note 1 and Lu et al. 2023 paper "Universal DNA methylation age across mammalian tissues" PMID: 37563227.
Wild animals	Samples from zoo-based animals were opportunistically collected and banked during routine health exams. This study also includes samples from wild animals, which were collected in the field: bats, deer. Details in Supplementary Note 1 and Lu et al. 2023 "Universal DNA methylation age across mammalian tissues" PMID: 37563227.
Reporting on sex	We profiled animals with both sexes, roughly half and half.
Field-collected samples	As the contributors are engaged in long-term field studies, care was taken to minimize disturbance during all the sample collections for different species. The details are described in species-specific papers.
Ethics oversight	We followed Institutional animal care and use protocols, or equivalent information from non-US contributors. All animals were maintained and bred under standard conditions consistent with National Institutes of Health guidelines and approved by the University of California, Los Angeles Institutional Animal Care and Use Committees. Details in Supplementary Note 1 and Lu et al. 2023 "Universal DNA methylation age across mammalian tissues" PMID: 37563227 See also the underlying species-specific papers published by the Mammalian Methylation Consortium. Non university organizations are certified either by the Associated Zoos and Aquariums (Lu bee Bat Conservancy) or by the Global Federation of Animal Sanctuaries (Bat World Sanctuary) or Elephant Taxon Advisory Group and Species Survival Plan.<br>All animals were maintained and bred under standard conditions consistent with National Institutes of Health guidelines and approved by the University of California, Los Angeles Institutional Animal Care and Use Committees. See also companion papers from the Mammalian Methylation Consortium (Haghani 2023 Science, Lu 2023 Nature Aging). Details on the protocol can be found in the Supplementary Note 1.

Note that full information on the approval of the study protocol must also be provided in the manuscript.
